# Targeting the signaling in Epstein–Barr virus-associated diseases: mechanism, regulation, and clinical study

**DOI:** 10.1038/s41392-020-00376-4

**Published:** 2021-01-12

**Authors:** Ya Cao, Longlong Xie, Feng Shi, Min Tang, Yueshuo Li, Jianmin Hu, Lin Zhao, Luqing Zhao, Xinfang Yu, Xiangjian Luo, Weihua Liao, Ann M. Bode

**Affiliations:** 1 Key Laboratory of Carcinogenesis and Invasion, Chinese Ministry of Education, Department of Radiology, Xiangya Hospital, Central South University, 410078 Changsha, China; 2 Cancer Research Institute and School of Basic Medical Science, Xiangya School of Medicine, Central South University, 410078 Changsha, China; 3 Key Laboratory of Carcinogenesis, Chinese Ministry of Health, 410078 Changsha, China; 4 Research Center for Technologies of Nucleic Acid-Based Diagnostics and Therapeutics Hunan Province, 410078 Changsha, China; 5 Molecular Imaging Research Center of Central South University, 410008 Changsha, Hunan China; 6 National Joint Engineering Research Center for Genetic Diagnostics of Infectious Diseases and Cancer, 410078 Changsha, China; 7 Department of Radiology, Xiangya Hospital, Central South University, 410078 Changsha, China; 8 The Hormel Institute, University of Minnesota, Austin, MN 55912 USA

**Keywords:** Cancer therapy, Cancer metabolism

## Abstract

Epstein–Barr virus-associated diseases are important global health concerns. As a group I carcinogen, EBV accounts for 1.5% of human malignances, including both epithelial- and lymphatic-originated tumors. Moreover, EBV plays an etiological and pathogenic role in a number of non-neoplastic diseases, and is even involved in multiple autoimmune diseases (SADs). In this review, we summarize and discuss some recent exciting discoveries in EBV research area, which including DNA methylation alterations, metabolic reprogramming, the changes of mitochondria and ubiquitin-proteasome system (UPS), oxidative stress and EBV lytic reactivation, variations in non-coding RNA (ncRNA), radiochemotherapy and immunotherapy. Understanding and learning from this advancement will further confirm the far-reaching and future value of therapeutic strategies in EBV-associated diseases.

## Introduction

Epstein–Barr virus was the first tumorigenic DNA virus that persistently infects human and some additional primate B cells. It exists as a free particle with a genome length of about 172 kb.^1^ EBV is transmitted primarily through saliva, resulting in asymptomatic infection in about 95% of the world’s population. In a variety of tumor types, EBV can integrate into the host genome at a remarkable rate to promote tumor development.^2^ As early as 1997, EBV was classified as a group I carcinogen by the International Agency for Research on Cancer (IARC) and its associated tumors account for 1.5% of all human cancers. The etiology of EBV is primarily relevant to malignancies of epithelial and lymphatic origin, including nasopharyngeal carcinoma (NPC), gastric cancer (GC), pulmonary lymphoepithelioma-like carcinoma (LELC), breast cancer, tonsillar cancer, hepatobiliary system cancer, malignant salivary gland tumors, and thyroid cancer, Hodgkin’s lymphoma (HL), non-Hodgkin’s lymphomas (NHL), including Burkitt’s lymphoma (BL), extranodal NK/T-cell lymphoma, and post-transplant lymphoproliferative carcinoma (PTLD).^3–5^ Recently, the National Institutes of Health (NIH) identified EBV as an essential element in reducing the global cancer burden.^5^ Moreover, EBV plays an etiological and pathogenic role in a number of non-neoplastic diseases, including infectious mononucleosis (IM), chronic active EBV infection (CAEBV), EBV-associated hemophagocytic lymphohistiocytosis (EBV-HLH), and other systemic autoimmune diseases, such as systemic lupus erythematosus (SLE), rheumatoid arthritis (RA), and Sjogren’s syndrome (SS).^6^

Notably, EBV infection of the host is predominantly latent. When the balance between host and virus is disturbed, EBV activation poses a risk of neoplasia. EBV has three latent infection types, type I is characterized by Burkitt’s lymphoma, which expresses only EBNA1 and the right fragment of BamHI A (BARTs) with the exception of EBERs. Type II includes NPC and Hodgkin’s lymphoma (HL) with EBV expressing EBNA1, LMP1, LMP2, BARTs, and EBERs. Type III includes lymphoproliferative diseases in immunosuppressed patients where EBV expresses all latent infection genes.^7^ LMP1 as a EBV encodes important oncogenic protein with specific functions occurring during latent infection and is essential for B-cell transformation in vitro.^8^ It mimics CD40 auto-oligomerization to continuously activate multiple intracellular signaling pathways that mediate various malignant phenotypes.^9^ LMP1 plays an important role in the development and progression of EBV-positive-associated tumors that are a prognostic marker for NPC patients.^10,11^ LMP2A and LMP2B were first discovered in 1990.^12^ LMP2A is a B-cell receptor (BCR) simulator that can protect BCR-depleted B cells from apoptosis.^13^ Epstein–Barr nuclear antigen 1 (EBNA1) is necessary to maintain stability of EBV particles, EBV replication,^14^ and also trigger B-cell lymphoma formation in transgenic mice.^15^ Studies have shown that EBNA1 causes genomic instability^16^ and promotes tumor cell survival.^17^ EBNA2 and EBNA3 are critical for B-cell transformation.^18^ Moreover, EBV has been shown to encode non-coding RNA,^19^ including BamHI-A rightward transcript microRNAs (miRNAs) (BART miRNAs), BHRF1 miRNAs, and EBV-encoded RNA 1 (EBER1) and EBER2, which interact with numerous proteins and facilitate the activation of innate immunity.

## EBV-associated diseases

### EBV and epithelial cancers

#### Nasopharyngeal carcinoma (NPC)

NPC is currently the most well-defined human epithelial cancer that is associated with EBV infection. In 1966, high titers of EBV-associated antibodies were found in NPCs.^20^ Studies have shown that an interaction of genetic, ethnic, and environmental factors may contribute to the development of NPC. In endemic areas, the EBV genome is detectable in almost all NPC tumor tissues. Subsequently, in the 1980s, serum levels of Epstein–Barr capsid antigen (VCA) immunoglobulin A (IgA) were established as one of the indicators for screening patients for NPC^21^ and the expression of latent genes in EBV-infected states was also discovered.^22^ With the use of qPCR to detect cell-free EBV DNA in 1999, a relationship between an increased risk of NPC due to EBV infection and poor prognosis was established.^23^ Since then, EBV DNA has become the gold standard for biological markers of NPC. Importantly, the genomic landscape of NPC has revealed that EBV latency genes provide growth and survival benefits, which result in the development of NPC. Therefore, with advances in NPC genomics, chromatin modifications enhance EBV load as a key feature of the NPC genome.^24^ Moreover, BALF2_CCT, as a high-risk subtype of EBV, promotes the progression of NPC in South China and facilitates early identification of high-risk individuals.^25^ In addition, Ephrin receptor A2 (EphA2), an epithelial growth factor-associated receptor, benefits EBV entry into epithelial cells.^26^ Importantly, common fragile regions are favored loci for EBV integration, which are genomic hotspots for DNA damage and vulnerable to genomic rearrangements. More recently, a study reports that EBV integration occur at 9.6% in NPC. These discoveries provide novel, potential intervention strategies for preventing EBV infection.

#### *EBV*-associated gastric cancer *(EBVaGC)*

Since 1992, the first demonstration of EBV in typical gastric adenocarcinomas occurred and facilitated the understanding of EBV in GC development.^27^ Approximately 9% of gastric cancer (GC) patients are EBV-positive, and these cases have a distinct molecular phenotype and clinical characteristics.^28^ Global epigenetic methylation and counteraction of the antitumor microenvironment are two major characteristics of this subtype of GC. With advances in the diagnosis and treatment of EBV infection, in situ hybridization (ISH) of EBER in biopsy specimens facilitate the diagnosis of EBVaGC.^29^ Recently, EBV can integrate into host genomes at 25.6% in GC tumorigenesis.^2^ In addition, immune checkpoint therapy for advanced stage or metastatic EBVaGC may be effective in treating this disease.^30^

#### Breast cancer

The first association of EBV infection with breast cancer was reported in 1995.^31^ Recently, several studies showed that EBV infection leads to more malignant transformation of breast epithelial cells and affects patient prognosis.^32,33^ EBV leads to a 4.74-fold increase in risk of breast cancer development compared with a control group.^32^ The potential oncogenesis mechanism of EBV is related to viral proteins, such as LMP1 that activate the Her2/Her3 signaling cascades.^33^

Several rare non-typical EBV-associated tumors, including pulmonary lymphoepithelioma-like carcinoma (LELC),^34^ tonsillar cancer,^35^ hepatobiliary system cancer,^36^ malignant salivary gland tumors,^37^ and thyroid cancer^38^ have also been reported. Among these, LELC is a rare and distinct subtype of primary lung cancer characterized by EBV infection and high LMP1 expression.^34^ These findings further expand our insight into the involvement of EBV in tumorigenesis.

### EBV and lymphatic system cancers

#### HL and NHL

EBV-associated lymphomas consist of HL and NHL, of which the most widely studied NHL are Burkitt’s lymphoma (BL) and extranodal NK/T-cell lymphoma.^39^ EBV particles were first detected in BL back in 1964.^40^ The EBV genome was next revealed in BL^41^ and the relationship between EBV and lymphoma was successfully determined epidemiologically in the 1970s.^42^ Moreover, the identification of EBV DNA in HL serum^43^ suggests that EBV may serve as a biomarker for early disease detection and monitoring patient treatment. In the 1980s, the EBNA protein was detected in HRS cells of a HL patient^44^ and then EBV genome was sequenced in HL and NK/T-cell lymphoma.^45^ Also, ENK/T-cell lymphoma was characterized by EBV infection of T or NK cells, which express a latency II pattern with variable LMP1 expression.

#### Post-transplant lymphoproliferative carcinoma (PTLD)

PTLD is a serious complication that occurs after solid organ, bone marrow, or blood stem cell transplantation, and is associated with EB virus infection in 60–80% of patients.^46^ In post-transplant patients, immunosuppression leads to repression of T-cells with a subsequent uncontrolled proliferation of lymphoid cells.^47^ With the discovery of the EBV genome and antibodies in 1980,^48^ a majority of PTLD patients have been tested for the presence of LMP1 and EBNA2/EBNA3 expression. The first successful use of EBV-specific T-cell therapy in EBV-associated lymphocyte proliferation occurred in 1995.^49^ Therefore, monitoring of EBV infection in post-transplant patients is of paramount importance.

### EBV chronic infectious diseases

#### Infectious mononucleosis (IM)

IM is a self-limiting disease caused by primary EBV infection, which was defined in 1968.^50^ Symptoms of IM are caused by an excessive immune response to EBV infection that presents with a “triad” of fever, pharyngitis, and lymphadenopathy, with a marked increase in peripheral blood lymphocytes and the appearance of heterogeneous lymphocytes.^51^

#### Chronic active EBV infection (CAEBV)

CAEBV is a serious EBV infection with clinical manifestations of persistent or recurrent IM-like symptoms.^52^ The main features of the advancement of the disease include dramatically enhanced levels of EBV DNA in the blood and infiltration of organs by positive EBER1-positive cells. Patients often exhibit fever, lymphadenopathy, splenomegaly, hepatitis, or pancytopenia.^53,54^ EBV infection of T cells and natural killer (NK) cells performs a pivotal contribution to the pathogenesis of CAEBV. Usually, T-cell infections have severe clinical symptoms and a poor prognosis. However, NK-cell-type infection patients favor for transformation into aggressive NK-cell leukemia or extranodal NK/T-cell lymphoma.^55^ Besides, in adult CAEBV, HLH may be the other clinical endpoint.^56–58^

#### EBV-associated hemophagocytic lymphohistiocytosis (EBV-HLH)

EBV-HLH is a severe, life-threatening hyperinflammatory syndrome of EBV infection, clinically characterized by abnormal proliferation and activation of lymphocytes and macrophages, fever, hepatosplenomegaly, blood cytopenias, hepatitis and/or hepatomegaly.^59^ Importantly, the timing and severity of these appearances can assist in distinguishing between EBV infection alone and HLH-complicated EBV infection.^60^

### EBV-related autoimmune diseases

EBV has been shown to be the cause of several autoimmune diseases, and studies have shown that EBV-infected individuals have a higher frequency of disease, including SLE, RA, SS, compared to non-infected individuals.^6^ As EBV plays an etiological role in autoimmune diseases, serological measures of EBV reactivation provide additional biomarkers to identify high-risk individuals. The common features of these EBV-related autoimmune diseases are elevated plasma viral loads and levels of EBV-directed antibodies that result in decreased EBV-directed cell-mediated immunity, suggesting the presence of widespread lytic EBV infection in patients.^61^ Furthermore, EBV reactive and the host’s immune response result in different diseases patterns and clinical manifestations.

## DNA methylation alterations and clinical applications in Ebv-associated malignancies

### Regulation of DNA methylation by EBV

DNA methylation in mammalian organisms is a reversible process, which mostly occurs by the addition of a methyl group to the C-5 position of cytosine in the CpG sequence context. DNA methyltransferases (DNMTs) transfer methyl groups from S-adenosyl-L-methionone (SAM) to cytosine and create 5-methylcytosine (5mC). DNMT1 is the most abundant DNMT in mammalian cells and it maintains methylation status during cell replication.^62^ Ten-eleven translocation methylcytosine dioxygenases (TETs) oxidize 5-mC to 5-hydroxymethylcytosine (5hmC), which in turn undergo further oxidation into 5-formylcytosine (5fC) and 5-carboxylcytosine (5caC), thereby mediating DNA demethylation.^63^ The balance of DNA methylation- demethylation dynamics is maintained by DNMTs and TETs.

**Fig. 1:**
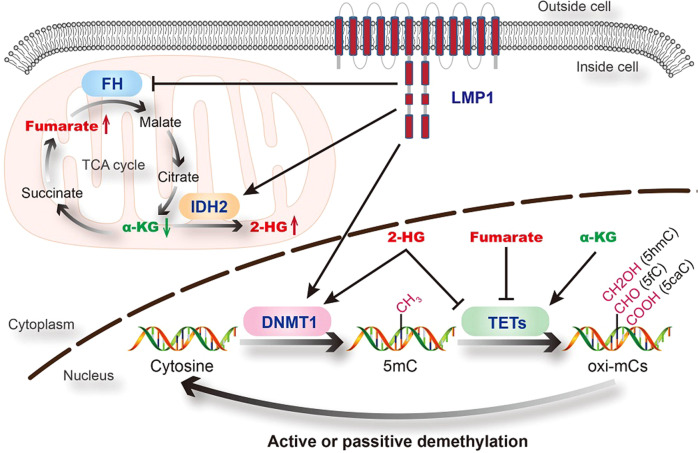
Regulation of DNA methylation by EBV (LMP1). The balance of DNA methylation- demethylation dynamics is maintained by DNMTs and TETs. DNMTs transfer methyl groups to cytosine and create 5mC. TETs iteratively oxidize 5mC to generate oxi-mCs (5hmC, 5fC, and 5caC). EBV (LMP1) enhances DNMT1 expression and activity, and also inhibit the enzymatic activity of TETs, which might tip the balance. FH fumarate hydratase, IDH2 isocitrate dehydrogenase 2

EBV infection is an epigenetic driver, which results in aberrant DNA methylation.^64,65^ First, EBV activates DNA methylation pathways. Tsai et al.^66^ reported that EBV-encoded latent membrane protein 1 (EBV-LMP1) enhances DNMT1 transcription and expression by activating the JNKs signaling pathway. We previously showed that EBV-LMP1 not only upregulates DNMT1 expression and activity, but also promotes its mitochondrial translocation.^67^ Secondly, EBV inhibits DNA demethylation pathway. TET family proteins, especially TET2, are downregulated during EBV infection, and TET2 may function as a resistance factor against EBV-induced DNA methylation acquisition.^68^ TET enzymes require α-ketoglutarate (α-KG) as an essential co-substrate to catalyze the conversion of 5-mC to 5-hmC. With a similar structure to α-KG, fumarate, succinate, and 2-HG could competitively inhibit the enzymatic activity of TETs.^69^ Recently, we revealed that EBV-LMP1 promotes the accumulation of fumarate and 2-HG, and the reduction of α-KG, which consequently leads to the inactivation of TETs.^70,71^ Moreover, 2-HG could also regulate DNMT1 activity by enhancing its binding to selected DNA regions.^72^ EBV infection might tip the balance in DNA methylation-demethylation dynamics (Fig. 1).

CpG island promoter methylation of tumor suppressor genes (TSGs) is one of the most characteristic abnormalities in EBV-associated malignancies. The CpG island methylation phenotype (CIMP) is defined as the high activity of global and nonrandom CpG island methylation. EBV infection mediates onco-epigenetic effects and is closely associated with CIMP.^73^ The most representative feature of EBV-positive GCs is an extensive hypermethylation phenotype, EBV-CIMP, which includes CDKN2A promoter hypermethylation.^74^ CIMP plays a driving role in EBV-associated malignancies, which leads to promoter hypermethylation and silencing of a series of functional genes (Tables 1–3).

**Table 1:** Hypermethylated genes in EBV-associated NPC

Gene names	Full name	Gene function	Methylation status	Reference
*APC*	*Regulator of WNT signaling pathway*	WNT signaling	34.0% (17/34)	^439^
*CADM1*	*Cell adhesion molecule 1*	Cell–cell adhesion	69.8% (37/53)	^83^
			67.9% (19/28)	^440^
*CDKN2A*	*Cyclin-dependent kinase inhibitor 2A, P16*	Cell cycle control	66.0% (35/53)	^83^
*CDH1*	*E-cadherin*	Cell–cell adhesion	73.7% (18/28)	^441^
*CDH13*	*Cadherin 13*	Cell–cell adhesion	77.4% (41/53)	^83^
*CHFR*	*Checkpoint with forkhead and ring finger domains*	Cell cycle control	58.5% (31/53)	^83^
*DAPK1*	*Death-associated protein kinase 1*	Apoptosis	27.3% (60/220)	^84^
*DAB2*	*DAB adaptor protein 2*	Proliferation	65.2% (30/46)	^442^
*DCC*	*DCC netrin 1 receptor*	Signal transduction	50.0% (23/46)	^439^
*DLEC*	*DLEC1 cilia and flagella-associated protein*	Proliferation	60.4% (29/48)	^439^
*DLC1*	*DLC1 Rho GTPase activating protein*	G-protein signaling	76.9% (40/52)	^83^
			43.8% (21/48)	^439^
*INPP4B*	*Inositol polyphosphate -4-phosphatase type II B*	Phosphatidylinositol signaling	60.0% (9/15)	^443^
*ITGA9*	*Integrin subunit alpha 9*	Cell–cell adhesion	55.6% (20/36)	^444^
*KIF1A*	*kinesin family member 1A*	Membrane trafficking	56.0% (28/50)	^439^
*MAOA*	*Monoamine oxidase A*	Amino acid metabolism	40.7% (22/54)	^445^
*miR-31*	*microRNA 31*	Post-transcriptional regulation	87.5% (14/16)	^446^
*PRDM2*	*PR/SET domain 2, RIZ1*	Histone methyltransferase	56.6% (30/53)	^83^
*PTEN*	*Phosphatase and tensin homolog*	PI3K/AKT signaling	80.0% (40/50)	^447^
*RARB*	*Retinoic acid receptor beta*	Nuclear receptor	15.9% (35/220)	^84^
*RASSF1A*	*Ras association domain family member 1*	Cell cycle control	24.1% (53/220)	^84^
			75.5% (40/53)	^83^
			82.1% (23/28)	^440^
*RASSF2A*	*Ras association domain family member 2*	Cell cycle control	29.2% (14/48)	^83^
*RRAD*	*Ras related glycolysis inhibitor and calcium channel regulator*	Calcium channel	74.3% (26/35)	^448^
*UCHL1*	*ubiquitin C-terminal hydrolase L1*	Protein ubiquitination	64.0% (32/50)	^439^
*WIF1*	*WNT inhibitory factor 1*	WNT signaling	51.8% (114/220)	^84^
			61.2% (30/49)	^83^
*WNT7A*	*Wnt family member 7A*	WNT signaling	69.4% (25/36)	^444^

**Table 2:** Hypermethylated genes in EBV-associated GC

Gene names	Full name	Gene function	Methylation status	Reference
*AQP3*	*Aquaporin 3*	Water channel	95.8% (23/24)	^449^
*B4GALNT2*	*Beta-1,4-N-acetyl-galactosaminyltransferase 2*	Blood group systems biosynthesis	50.8% (32/63)	^450^
*CDKN2A*	*Cyclin-dependent kinase inhibitor 2A, P16*	Cell cycle control	86.7% (13/15)	^451^
			68.2% (15/22)	^452^
*CDKN2B*	*Cyclin-dependent kinase inhibitor 2B, P15*	Cell cycle control	86.7% (13/15)	^451^
*CDH1*	*E-cadherin*	Cell–cell adhesion	100% (15/15)	^451^
*CYLD*	*CYLD lysine 63 deubiquitinase*	Deubiquitination	56.0% (14/25)	^453^
*DAPK1*	*Death-associated protein kinase 1*	Apoptosis	73.3% (11/15)	^451^
*IRF5*	*Interferon regulatory factor 5*	Transcription factor	76.5% (13/17)	^454^
*MARK1*	*Microtubule affinity regulating kinase 1*	Cell migration	68.0% (17/25)	^455^
*MINT2*	*amyloid beta precursor protein binding family A member 2*	Signal transduction	92.0% (23/25)	^452^
*PTEN*	*Phosphatase and tensin homolog*	PI3K/AKT signaling	64.3% (18/28)	^456^
*REC8*	*REC8 meiotic recombination protein*	Meiosis	92.3% (12/13)	^457^
*RUNX3*	*RUNX family transcription factor 3*	Transcription factor	96.0% (24/25)	^452^
*S100A4*	*S100 calcium binding protein A4*	Signal transduction	50.0% (12/24)	^455^
*SCRN1*	*Secernin 1*	Exocytosis	48% (12/25)	^455^
*SOX9*	*SRY-box transcription factor 9*	Transcription factor	85.7% (6/7)	^458^
*SSTR1*	*Somatostatin receptor 1*	G-protein-coupled receptor signaling	75% (9/12)	^459^
*ST3GAL6*	*ST3 beta-galactoside alpha- 2,3- sialyltransferase 6*	Glycosphingolipids biosynthesis	44.4% (28/63)	^450^
*TP73*	*Tumor protein p73*	Stress response	96.0% (24/25)	^455^
			100% (15/15)	^451^
			96.0% (24/25)	^452^
*WNT5A*	*Wnt family member 5A*	WNT signaling	91.3% (21/23)	^460^

**Table 3:** Hypermethylated genes in other EBV-associated tumors

Gene names	Full name	Gene function	Cancer type	Methylation status	Reference
*BCL2L11*	*BCL2 like 11*	Apoptosis	BL	92.9% (13/14)	^461^
*CDKN2A*	*Cyclin-dependent kinase inhibitor 2A, P16*	Cell cycle control	CSCC	61.0% (25/41)	^462^
*CDH1*	*E-cadherin*	Cell–cell adhesion	OSCC	82.4% (56/68)	^463^
*HACE1*	*HECT domain and ankyrin repeat containing E3 ubiquitin protein ligase 1*	Protein ubiquitination	NKTCL	100% (6/6)	^464^
*MGMT*	*O-6-methylguanine-DNA methyltransferase*	DNA repair	OSCC	55.9% (38/68)	^463^
*PRDM1*	*PR/SET domain 1*	Transcriptional regulation	BL	50.0% (11/22)	^465^

*BL* Burkitt’s lymphoma, *CSCC* cervical squamous cell carcinoma, *OSCC* oral squamous cell carcinoma, *NKTCL* natural killer/T-cell lymphoma

Interestingly, DNA hypomethylation induced by EBV has also been observed in a few studies. For example, the S100 calcium binding protein A4 (S100A4) is overexpressed in LMP2A-positive NPC tissues and DNA hypomethylation plays a critical role in S100A4 regulation.^75^ In Hodgkin’s lymphoma, a set of tumor-related genes known to be silenced by promoter hypermethylation (e.g., *RASSF1, DAPK, MGMT*) are more frequently hypermethylated in EBV-negative compared to EBV-positive cases.^76^ EBV might also take part in the redistribution of DNA methylation modification sites.

### The clinical significance of DNA methylation as a biomarker in EBV-associated malignancies

Abnormal DNA methylation has been investigated in NPC biopsies, nasopharyngeal brushings, and blood samples. Thus, aberrant DNA-methylated genes might be used as diagnostic or prognostic biomarkers.

In the EBV genome, DNA methylation takes an important part in lytic cycle regulation. The EBV genome is gradually methylated during the establishment of latency, while it becomes unmethylated in the viral lytic cycle. EBV DNA methylation could be used as a biomarker. For example, Zheng et al.^77^ found that a combination of the degree of EBV DNA methylation and EBV DNA load showed great potential for diagnosing NPC in nasopharyngeal brushing samples (sensitivity = 0.98, specificity = 0.95). The viral genome-wide methylation profiling of circulating EBV DNA in plasma demonstrated differential methylation patterns between NPC and non-NPC subjects. When combining the plasma EBV DNA load and methylation test pattern in plasma samples, the sensitivity was 0.97 and the specificity was 0.99 for population screening of NPC.^78^

Tumor-related genes are associated with EBV infection and carcinogenesis and some of these are summarized in Table 1. For instance, *RASSF1A* promoter methylation is a promising noninvasive biomarker for the diagnosis of NPC as determined from tissue and brushing samples (tissue: sensitivity = 0.72, specificity = 0.99, AUC = 0.98; brushing: sensitivity = 0.56, specificity = 1.00, AUC = 0.94).^79^ High levels of *TIPE3* promoter methylation is associated with worse overall survival (OS), disease-free survival (DFS), and distant metastasis-free survival (DMFS), which could be an independent prognostic factor in NPC.^80^ The methylation level of the *HOPX* promoter is a potential prognostic biomarker for NPC and high levels are associated with poor prognosis.^81^ Patients with *RAB37* hypermethylation are more sensitive to docetaxel-containing induction chemotherapy and *RAB37* methylation levels might be used to predict chemotherapy sensitivity.^82^

Recently, panels of methylation genes, which are more sensitive than single biomarkers, have been reported. Hutajulu et al.^83^ found that combined analyses of five methylation markers (i.e., *RASSF1A, p16, WIF1, CHFR, RIZ1*) provided good discrimination between NPC and non-NPC patients with a detection rate of 98%. A panel of four methylated genes (i.e., *RASSF1A, WIF1, DAPK1, RARβ2*) in combination with the EBV DNA test increase the sensitivity for NPC detection at an early stage (sensitivity = 0.96, specificity = 0.65, AUC = 0.83).^84^ The genome-wide DNA methylation profiling study revealed a methylation gene panel (i.e., *WIF1, UCHL1, RASSF1A, CCNA1, TP73, SFRP1*) as a prognostic biomarker for OS and DFS, which was also associated with the efficacy of concurrent chemotherapy in NPC.^85^

### Targeting DNA methylation for therapy against EBV-associated malignancies

DNA hypermethylation correlates with poor survival outcomes in patients with cancer, especially with EBV-associated tumors. DNMT1 (maintaining DNA hypermethylation of specific genes) is currently the most important target for epigenetic cancer therapy. DNMT inhibitors, such as Vidaza^®^ (5-azacytidine, Celgene) and Dacogen^®^ (decitabine or 5-aza-2’-deoxycytidine, SuperGen) have been approved by the FDA for cancer therapy.^86^ A few studies have focused on EBV-associated malignancies. In NPC preclinical studies, 5-azacytidine has shown the potential to improve outcomes with radiotherapy.^87^ However, in a phase II study, CC-486 (oral azacitidine) did not show sufficient clinical activity to support further development as a monotherapy in advanced NPC.^88^

Some natural compounds also showed efficacy as DNMT1 inhibitors. Previous studies from our group and other labs have reported that a farnesyl phenolic compound, grifolin, isolated from the fresh fruiting bodies of the mushroom Albatrellus confluens, exhibits extensive bioactivity.^67,89–95^ Most recently, we demonstrated that grifolin treatment inhibits DNMT1 expression and activity, as well as its mitochondrial translocation. Consequently, it results in demethylation of the *pten* gene and inhibition of downstream AKT signaling, which effectively attenuates glycolytic flux in NPC cells. Moreover, demethylation of the mtDNA D-loop region and upregulation of oxidative phosphorylation (OXPHOS) complexes are observed with grifolin treatment in NPC cells.^67^

## Metabolic reprogramming and clinical application to Ebv-associated cancers

### Glycolysis in NPC

Enhanced glucose metabolism is a common hallmark of cancer. Virus can increase aerobic glycolysis and use glucose biosynthetically.

EBV infection alters glucose metabolism and cause the Warburg Effect. For example, EBV upregulation of all glycolytic enzymes including rate-limiting enzyme hexokinase 2 (HK2), also EBV infection induced substantial relocalization of glucose transporter-1 (GLUT-1) and enhanced glycolytic flux. Elevated glycolysis has been demonstrated by EBV oncoprotein LMP1.^96^ An earlier study showed that GLUT-1 is highly expressed in the nasopharyngeal tissues of patients with NPC, and its expression is associated with clinical stage, lymph node metastasis, and EB virus infection.^97^ Some researchers suggested that Epstein–Barr virus-encoded LMP1 changes the metabolic profile and promotes increased glycolysis in NPC cells.^98,99^ According to Xiao et al.,^98^ a large-scale metabolic profiling approach showed that EBV (LMP1) regulated metabolic changes in NPC cells and, in particular, the promotion of glycolysis. HK2 was identified as a key modulator of LMP1-induced glycolysis, and conferred proliferative advantages and poor prognosis of NPC patients following radiation therapy. The LMP1-perturbed PI3-K/Akt-GSK3β-FBW7 signaling axis resulted in the stabilization of c-Myc, which was confirmed to be crucial for LMP1-induced glycolysis and confers NPC cells resistance to apoptosis.^98^ Lu et al.^99^ revealed a novel mechanism for LMP1-mediated radio-resistance by suppressing the DNA damage response (DDR) through DNA-PK/AMPK signaling. LMP1 reduced the phosphorylation of AMPK and disturbed its subcellular localization after irradiation. The findings showed that inhibition of AMPK signaling also accelerated glucose uptake and lactate production and conferred resistance of NPC cells to apoptosis induced by irradiation. Reactivation of AMPK by metformin, a pharmacological activator of AMPK, could substantially reverse radio-resistance of LMP1-positive NPC cells both in vitro and in vivo*.*^99^ EBV epigenetically reprogram several Hox genes expression in NPC cell lines and tumor biopsies. Ectopic expression of HoxC8 inhibits NPC cell growth in vitro and in vivo, modulates glycolysis and regulates the expression of TCA cycle-related genes.^100^ Recently, Luo et al.^67^ analyzed the metabolites of ^13^C6-d-glucose-traced NPC cells by using gas chromatography/mass spectrometry (GC/MS), and found that glycolytic flux in LMP1-overexpressing cells was markedly increased by about 90% compared to that in LMP1-negative cells. In addition, ^13^C-labeled pyruvate and lactate levels were markedly elevated, whereas ^13^C-labeled metabolite levels of the tricarboxylic acid cycle, including ^13^C-citrate, ^13^C-α-ketoglutarate, ^13^C-fumarate, and ^13^C-malate were significantly decreased.^67^ Importantly, our latest findings revealed that LMP1-positive NPC cells have a powerful mitochondrial fission function to sustain metabolic changes. Moreover, the phosphorylation of mitochondrial dynamin- protein Drp1 (Ser616 or Ser637) was indispensable for the increased glycolytic metabolism regulated by LMP1. The enhancement of glycolysis might be a crucial adaptive function of cancer cells under the high fission state of mitochondria induced by EBV infection stress.^101^ Increasing evidence suggests the progression of tumors might not only rely on the cancer cells themselves but also be influenced by the surrounding stroma cells. Extracellular vesicles packaged LMP1 activated normal fibroblasts (NFs) to cancer-associated fibroblasts (CAFs) via the NF-κB p65 signaling pathway, which promote tumor progression via autophagy and stroma-tumor metabolism coupling.^102^

Fibroblast growth factor 1 (FGFR1) signaling is a key pathway in LMP1-mediated growth transformation. LMP1-mediated FGFR1 activation contributes to aerobic glycolysis and transformation of epithelial cells, thereby implicating FGF2/FGFR1 signaling activation in the EBV-driven pathogenesis of NPC.^103^ EBV-LMP1 induces glycolytic addiction to enhanced cell motility in nasopharyngeal epithelial cells. EBV-LMP1 activates IGF1-mTORC2 signaling and nuclear acetylation of the Snail promoter by PDHE1α, an enzyme involved in glucose metabolism, to enhance cell motility, thereby driving cancer metastasis. The IGF1/mTORC2/PDHE1α/Snail axis correlates significantly with disease progression and poor prognosis in NPC patients.^104^

EBV infection and enhanced glucose metabolism might drive the development and progression of EBV-associated cancers. These findings provide a new perspective to reveal for the interplay between EBV infection and host metabolism-associated molecules in driving cancer pathogenesis. Understanding these key events might reveal novel therapeutic targets and potentially reverse resistance to treatment therapies in EBV-associated cancers patients.

### Targeting glycolysis by natural compounds

Natural compounds derived from plants, fungi, and marine organisms are widely used as anticancer drugs in preclinical/clinical development or in clinic.^105^ A number of natural compounds that target metabolism-related pathways show inhibition activity on cancer cells.^106^ Neoalbaconol (NA), isolated from the fruiting body of the mushroom Albatrellus confluens, is a novel small-molecular compound with a drimane-type sesquiterpenoid structure.^107^ In response to various stimulations, PDK1 can activate phosphoinositide-3 kinase (PI3K) and then Akt, playing pivotal roles in energy metabolism, cell proliferation and migration. By using EBV-positive C666-1 cell line, we found that neoalbaconol inhibits phosphorylation of Akt and downstream targets, including TSC2, mTOR, and p70S6K1.^108^ Also, neoalbaconol inhibited the activity of key energy metabolic enzyme hexokinase 2 (HK2), which eventually resulted in a striking energy crisis. Furthermore, neoalbaconol treatment not only significantly decreased the glucose concentration in the media but also blocked ATP generation.^108^ Seahorse XFp cell energy phenotype test showed that cell oxygen consumption rate (OCR) and extracellular acidification rate (ECAR) were decreased after neoalbaconol treatment. In addition, by using a nude mouse model, we found that neoalbaconol decreases C666-1 cell xenograft tumor growth and suppresses the PI3K/Akt pathway in vivo*.*^108^ In conclusion, neoalbaconol regulates PDK1/Akt/mTOR/p70S6K1 signaling pathway and glycolytic gene hexokinase, thus decreasing cell glycolysis and ATP synthesis to induce NPC cell death and inhibit tumor growth in vivo. This study is expected to contribute to the rational design and critical assessment of novel anticancer therapies based on natural compounds-mediated glycolysis reprogramming in EBV-related cancer.

### Lipid alteration and fatty acid oxidation in EBV-associated cancers

Aberrant Lipid reprogramming is involved in promoting tumor progression and membrane homeostasis. EBVaGC has a different phospholipid and triacylglycerol (TG) expression pattern than EBV-negative gastric cancer. Fifteen of these lipid-related pathways were associated with EBVaGC metabolites. Analysis of the three major lipids, phospholipids, sphingolipids and neutral lipids revealed that TG and ceramide were downregulated, whereas phosphatidylcholine (PC) and phosphatidylethanolamine (PE) were upregulated in EBVaGC. These findings may help explain the mechanism of EBVaGC tumorigenesis.^109^ Furthermore, we know that in primary nasopharyngeal carcinoma, EBV-LMP1 induces lipid synthesis and lipid droplet formation. Lo et al.^110^ reported a novel function of LMP1 in promoting nascent lipogenesis. EBV-LMP1 increased the expression, maturation, and activation of steroid regulatory element-binding protein 1 (SREBP1) and its downstream target fatty acid synthase (FASN). In addition, elevated FASN expression was associated with poor survival in patients with invasive disease and NPC. Their findings suggest that LMP1 activation of SREBP1-mediated adipogenesis promotes tumor cell growth and is involved in the EBV-driven pathogenesis of NPC, which propose a new therapeutic protocol for the treatment of locally advanced or metastatic nasopharyngeal carcinoma using lipogenesis inhibitors.

Fatty acids are fundamental cellular components and energy sources. Fatty acid oxidation (FAO) is increasingly recognized as a crucial metabolic characteristic of cancer. For example, a group of the OXPHOS cluster diffuse large B-cell lymphomas (OXPHOS-DLBCL), which is significantly enriched in genes involved in mitochondrial oxidative phosphorylation, predicts potential differences in mitochondrial oxidative metabolism compared with other DLBCL groups. Notably, palmitate is a predominant respiratory fuel in OXPHOS-DLBCLs. NADPH and ATP is mainly supplied through the fatty acid β-oxidation and coupled oxidative phosphorylation, providing growth and proliferation advantages.^111^

**Fig. 2:**
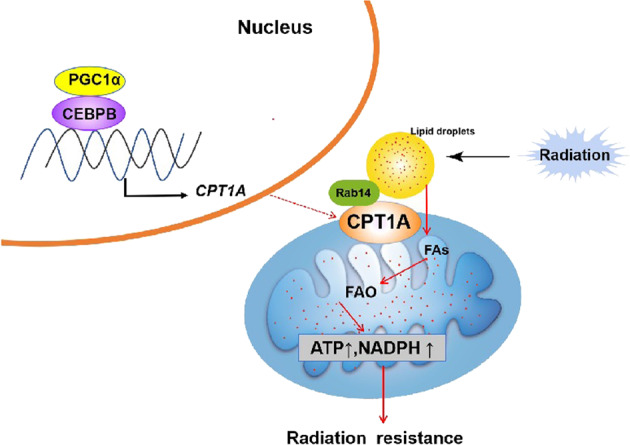
A schematic to illustrate CPT1A-mediated radiation resistance in NPC. PGC1α-CEBPB interaction promote the transcription, expression and enzyme activity of CPT1A, and CPT1A-Rab14 interaction promote fatty acid trafficking between lipid droplets and mitochondria, which facilitates fatty acid utilization and maximizes ATP and NADPH production, leading to resistance to radiation

Moreover, lipid reprogramming may play a vital role in mediating radiation resistance in NPC (Fig. 2). An active FAO is a vital signature of NPC radiation resistance, and the rate-limiting enzyme of FAO, carnitine palmitoyl transferase 1 A (CPT1A), was consistently upregulated in these cells.^112^ The PGC1α-CEBPB interaction promotes CPT1A transcription, which can facilitate FAO, and maximizes ATP and NADPH production, contributing to radiation resistance.^113^ Furthermore, the IHC scores in a tissue microarray indicated that PGC1α was highly expressed in non-keratinizing undifferentiated NPC, which corresponds to worse overall survival after radiation therapy in NPC.^113^ More interestingly, the CPT1A-Rab14 interaction plays roles in CPT1A-mediated radiation resistance by facilitating fatty acid trafficking. Inhibition of CPT1A resensitized NPC cells to radiation therapy by activating mitochondrial apoptosis both in vitro and in vivo*.*^112^ Targeting the PGC1α/CEBPB/CPT1A/FAO signaling axis by etomoxir (ETO) or genetic knockdown could be a potential strategy to improve the therapeutic effects of radiotherapy in NPC.^113^ Mechanistic studies of abnormal tumor fatty acid metabolism and radiotherapy resistance will help enrich our understanding of tumor lipid metabolism reprogramming and provide new perspectives and novel molecular targets for tumor radiotherapy resistance. However, how the EBV plays a role in this FAO need further exploration.

### Glutamine metabolism in NPC

Glutamine is one of the important precursors contributing to *de novo* nucleotide synthesis, which provides the nitrogen that is required for purine and pyrimidine nucleotide synthesis. Glutamine synthetase (GS) is responsible for glutamine metabolism in cancer therapeutic responses, in particular under irradiation-induced stress. GS is transcriptionally regulated by STAT5 and highly expressed in radiation-resistant NPC cells. High expression of GS drives metabolic flux toward nucleotide synthesis to enable efficient DNA repair, then acquired radiation resistance and survival advantages. Targeting GS and the associated pathways may provide therapeutic benefits to patients with both intrinsic and acquired radiation resistance.^114^

### One-carbon metabolism in EBV-induced B-cell transformation

One-carbon (1C) has a critical contribution to speedy cell growth in support of embryonic progression, cancer and T-cell activation. Mitochondrial 1C metabolism stands out as one of the highest induced pathways for EBV infection. EBV triggers mitochondrial 1C metabolism in newly infected B cells, and 1C upregulation is necessary for efficient growth, survival, and transformation of these cells. Besides, EBV induced mitochondrial folate metabolism enzyme methylenetetrahydrofolate dehydrogenase 2 (MTHFD2), which was driven transcriptionally by EBV-encoded protein EBNA2 and MYC synergistically. EBV-induced mitochondrial 1C metabolism drives nucleotide, mitochondrial NADPH, and glutathione production. Which provide an attractive rationed for developing novel mitochondrial 1C metabolic inhibitors for the treatment of B lymphomas.^115,116^

### Clinical application of metabolic analysis in EBV-related cancers

#### Serum metabolic profiling and radiotherapy in NPC

Based on gas chromatography-mass spectrometry (GC-MS) metabolomics coupled with partial least squares-discriminant analysis, an NPC discrimination model was established with a sensitivity of 88% and a specificity of 92%. Seven metabolites, including glucose, linoleic acid, stearic acid, arachidonic acid, proline, β-hydroxybutyrate, and glycerol 1-hexadecanoate, were identified as contributing most to the discrimination of NPC serum from healthy controls. Metabolic footprints of 20 NPC patients treated with standard radiotherapy are visually submitted to validate the model applied for therapeutic evaluation. The coincident rate of the trends of metabolic footprints to the actual clinical prognosis trend was ~80%. The metabolic profiling approach as a novel strategy may be capable of delineating the potential of metabolite alterations in discrimination and therapeutic evaluation of NPC patients.^117^

#### ^18^F-FDG-based imaging in EBV-associated tumors

^18^F-Fluorodeoxyglucose (^18^F-FDG) positron emission tomography (PET), which identifies viable tumors by detecting enhanced tumor glycolysis, has long been used as an imaging modality for staging NPC and detecting recurrence of the disease. HK2 has been recognized as a therapeutic target. Two NPC clinical investigations showed that high ^18^F-FDG uptake indicates poor outcome in patients with NPC^118,119^, which is highly dependent on HK2. Xiao et al.^98^ suggested that therapeutic strategies targeting multifunctional metabolic genes, such as HK2, would provide more effective treatment options for NPC.

Previously, associations of combined ^18^F-FDG-PET and MRI parameters with histopathological features depended on tumor grading and correlated strongly with tumor cell expression of Ki-67 and HIF-1α in head and neck squamous cell carcinoma (HNSCC).^120^ Also, this metabolic imaging biomarker-based parameters are useful for evaluation of prognostic significance. Maximum and mean standardized uptake values (SUVmax, SUVmean) and total lesion glycolysis (TLG) of the primary tumor could predict a poor outcome in NPC patients.^121^

Recently, the plasma EBV DNA titer has been recognized as a particularly promising prognostic biomarker. For instance, investigations have compared the prognostic value of ^18^F-FDG PET parameters and the EBV DNA titer during radiotherapy in patients with primary M0 NPC. Combining ^18^F-FDG-PET- derived parameters and EBV DNA to subtype patients with primary NPC is more beneficent than the conventional TNM system. Furthermore, ^18^F-FDG PET-derived parameters and the EBV DNA titer can predict outcome in patients with primary NPC, which may represent an imaging-guided and biomarker-guided double evaluation strategy in the future.^122^

Thus, all the evidences point out that EBV infection effectively disrupts cellular metabolic homeostasis, and understanding these mechanism-based metabolic reprogramming-induced EBV will reveal new avenues for future diagnosis and treatment.

## Mitochondria and Ebv-associated tumors

**Fig. 3:**
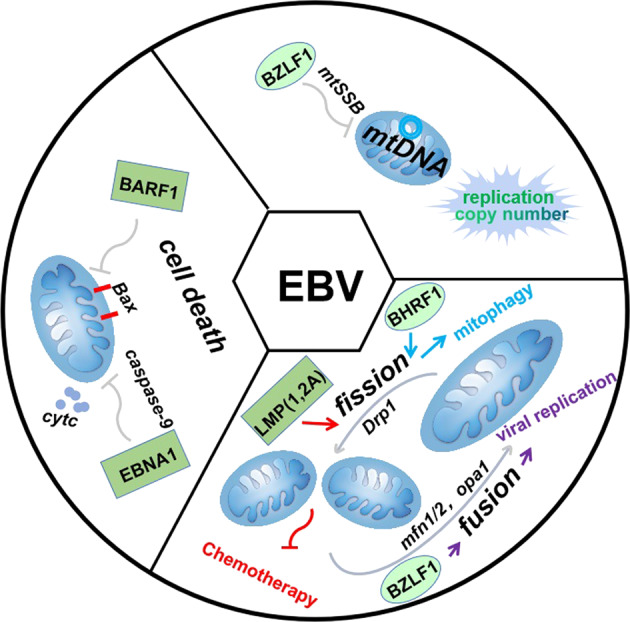
Mitochondria play an important etiological and pathogenesis role in the EBV-associated cancers. EBV has a new function in remodeling of mitochondrial dynamics during latency and lytic infection. EBV-encoded latent oncogene BARF1 and EBNA1 suppress the apoptotic pathways by decreasing Bax translocation to mitochondria and activating casepase-9. The BZLF1 protein encoded during EBV lytic activation inhibits the replication and copy number of mtDNA

Mitochondria play an important role in cellular energy dynamics, and are the hubs that connect numerous signaling pathways in tumors. Mitochondrial DNA, mitochondrial fission and fusion dynamics, mitochondrial-related cell death are involved in coordinated processes of cellular physiological and pathological regulation, highlighting the multidimensional function of mitochondria in cancers (Fig. 3).

### Mitochondria DNA (mtDNA)

#### Mitochondria DNA (mtDNA) and NPC

Mitochondrial biosynthesis and function are, of course, largely controlled by mitochondrial DNA. Ethidium bromide can consume mitochondrial DNA and inhibit the proliferation of tumor cells, indicating that mitochondria are important contributors to tumor growth and invasion.^123^ One early study demonstrated that mutations in mitochondrial DNA may be involved in the development and progression of NPC. Mutations in mitochondrial DNA are mainly caused by the deletion of its common deletion (CD) of 4981 bp, the accumulation of which in patients might be involved in the development and progression of NPC linked with patient age and clinical stage.^124^ The mitochondrial genome is also considered to be one of the genetically susceptible areas in NPC. Research has indicated that NPC patients often exhibit higher mtDNA mutation frequency compared to normal nasopharyngeal epithelial tissues. Patients with NPC showed a high frequency of mutation at mtDNA np16362, np16519, and mtMSI D310 and have family heritability.^125^ The effect of mtDNA haplogroup on NPC occurrence was evaluated in 201 NPC patients with matched controls. Results indicated that patients with haplogroup R9, and its sub-haplogroup F1, in particular, exhibited the most vigorous development of NPC.^126^

#### Mitochondria DNA (mtDNA) and EBV

Mitochondria have been known to serve as targets for tumor virus aggression and also to intervene in viral tumorigenesis. BZLF1 is a DNA binding protein belonging to the basic leucine zipper transcription factor family.^127^ MtDNA replication is initiated by transcription-mediated priming facilitated by the mitochondrial single-stranded DNA binding protein (mtSSB). MtSSB is a tetramer composed of four 16 kDa subunits and binds to mitochondrial DNA at the transcription and replication initiation area.^128^ BZLF1 targets mtSSB and mediates the translocation of mtSSB from mitochondria into the nuclear compartment, thus inhibiting mtDNA replication, and decreasing mtDNA copy number.^129^

### Mitochondrial dynamics triggered by EBV

Mitochondrial dynamics mainly consist of two mutually restrictive shaping processes: mitochondrial fission and fusion.^130^ Under severe stress, mitochondria tend to divide and exhibit rounded, fragmented morphology. When subjected to mild stimuli, mitochondria predominantly show a rod-shaped fused morphology.^131^ At present, mitochondrial dynamics have been used to classify tumors, predict clinical prognosis, and therapeutic response. The key regulators involved in mitochondrial remodeling proteins include mitochondrial dynamin-related protein (Drp1), mitochondrial outer membrane receptor proteins (Fis1, Mff, mid49/51) and mitochondrial fusion proteins (Mfn1/2, OPA1). These shaping proteins contribute more to mitochondrial function and cell metabolism.^132^ Drp1 is a factor in mitochondrial dynamics that is involved in tumor migration and pathogenesis.^133–135^ Crucially, phosphorylation of Drp1 has a vital role in the modulation of Drp1 activity. Increasing evidence indicates that Ser616 phosphorylation facilitates the movement of Drp1 from the cytoplasm to the outer mitochondrial membrane. However, Ser637 phosphorylation causes a reversal of this procedure.^136^

#### Mitochondrial dynamics and EBV latency infection

Mitochondrial heterogeneity may be responsible for the recurrence and treatment resistance of EBV-associated tumors.^67,124^ EBV encodes the oncogene LMP1 and latent membrane protein 2A (LMP2A), which play important roles in gene expression and are thought to be involved in EBV-mediated tumorigenesis.^137^ EBV expresses oncogenic proteins that can interfere with the function of CDKs and induce a number of signals that put their genomes replicate into the host’s cells.^138^ Recently, we revealed that CDK1 binds to Drp1 in the mitochondria to form a complex that enhances mitochondrial fission. Also, EBV-LMP1 has a novel function in mitochondrial remodeling as evidenced by enhanced mitochondrial fission and glycolytic metabolism through regulation of the cyclin B1/Cdk1/Drp1 signaling axes. This study implies that changes in mitochondrial morphology caused by EBV- encoded oncogenes promote mitochondrial function and reprogramming metabolism.^101^ Another latent protein, LMP2A, has also been reported to promote mitochondrial fission, induce cell migration, and epithelial–mesenchymal transition (EMT) in gastric and breast cancer cells, suggesting that EBV-driven mitochondrial fission promotes tumor cell survival and mediates drug-resistant therapy.^139^

#### Mitochondrial dynamics and EBV lytic infection

Zta (BZLF1 or EB1) and Rta (BRLF1) are immediate early response proteins, which are expressed at the early stage of EBV infection and have been reported to reorganize mitochondrial morphology during EBV lytic replication. Interestingly, a fusion event initiated by BZLF1 expression and the expression of BRLF1 resulted in the relocalization of mitochondria to one side of the nucleus.^140^ Furthermore, BHRF1 is expressed during the viral productive cycle and is able to translocate to mitochondria and shows strong functional homology with the human Bcl2 protein. BHRF1 prevents type I IFN pathway activation by encouraging mitochondrial fragmentation, which ultimately contributes to autophagosomes degradation by mitophagy.^141^ Thus, the loss of control of the EB virus stress over mitochondrial structure will be another checkpoint to overcome.

### Targeting mitochondrial signaling

#### Targeting mitochondrial fission or fusion

Proteins involved in mitochondrial fusion and fission may participate in cancer cell resistance to apoptotic stimuli and serve as new therapeutic targets. Based on the observed high fission-to-fusion ratio in apoptotic cells, fission is believed to be a necessary prerequisite for apoptosis.^142^ The TP53-regulated inhibitor of apoptosis (TRIAP1) was discovered to be aberrantly overexpressed and associated with poor survival in NPC patients. Furthermore, miR-320b is a negative regulator of TRIAP1 and upregulation of miR-320b suppresses NPC cell proliferation and encourages mitochondrial fission and apoptosis.^143^ 1,8-Dihydroxy-3-acetyl-6-methyl-9,10 anthraquinone (GXHSWAQ-1) targets mitochondrial proteins (CDH1, RAC1, CDC42) to enhance radiosensitization by altering the mitochondrial structure, including swollen volume, fragmented crista, and decreasing transmembrane potential.^144^

Furthermore, contrasting evidence points to Drp-mediated mitochondrial fission as an anti-cell death factor. Most recently, Drp1-regulated mitochondrial fission not only promotes the proliferation of tumor cells, but also participates in the maintenance of cell stemness, affecting tumor invasion and metastasis as well as tumor treatment.^145^ One study has shown that inhibiting Drp1 activity by interrupting the Cox-2/Drp1 axis, reducing the stemness of NPC cells, and helping to increase its sensitivity to resveratrol and 5-flumidine treatment,^146^ suggesting that targeting Drp1-dependent mitochondrial fission would be an effective treatment for NPC. In fact, the involvement of Drp1 in tumor pathogenesis and therapeutic sensitization was also confirmed in our report, demonstrating that mitochondrial fission can be used to classify cancers and play an important role in predicting clinical prognosis and therapeutic response of NPC. Notably, the protein level of p-Drp1 (Ser616) was positively related to the clinical stage of NPC. Patients with a high level of p-Drp1 (Ser616) showed shorter DFS and OS. In contrast, a high level of p-Drp1 (Ser637) in NPC patients is associated with a longer DFS and OS, suggesting that either serine 616 or serine 637 phosphorylation of Drp1 is a prognostic biomarker of NPC patients. What makes more sense is that metformin interrupts the AMPKɑ/ p-Drp1 Ser637 signaling pathway or cucurbitacin E targets the cyclinB1-Cdk1/p-Drp1 (Ser616) axis repressing mitochondrial fission.^101^ This discovery supports the mitochondrial dynamics markers Drp1 Ser616 and Ser637 as crucial targets for NPC in the future.

#### Targeting mitochondrial induces apoptosis

Previous studies focusing on the regulation of mitochondria in apoptosis indicated that pro-apoptotic proteins translocate to the mitochondrial outer membrane and trigger the source of reactive oxygen species (ROS) and apoptosis directly.^147–149^ Interrupting mitochondrial function to induce apoptosis may be a novel mechanism for targeted EBV-associated tumor therapy.

EBV-encoded oncogene *BARF1* (*BamH1-A Rightward Frame-1*) is a survival factor by suppressing apoptosis pathways through modulation of the Bcl-2/Bax ratio. Knock down of BARF1 by siRNA induces mitochondrial-dependent cell death.^150^ In addition, Epstein–Barr nuclear antigen 1 (EBNA1) is the only protein expressed in all three types of latent infection, is also involved in the resistance to mitochondrial-dependent cell death. Triptolide, a diterpene epoxide of the tristeza extract, has been shown to target EBNA1, inhibit proliferation of EBV-positive NPC cells, and induce cell death through a caspase-9-dependent pathway.^151^ For example, extranodal natural killer/T-cell lymphoma (ENKTL) is characterized by EBV latent infection with the expression of EBNA1 and LMP1. Importantly, targeting hexokinase domain component 1 (HKDC1), which is highly expressed in ENKTL, is associated with mitochondrial dysfunction and oxidative stress, EBV replication, and P-glycoprotein (P-gp) expression.^152^ The above studies further clarify the role of mitochondria-related cell death in the EBV tumorigenesis process, which will help us to fully understand the molecular mechanism of mitochondria in the pathogenesis of EBV-related tumors, and will be beneficial to the discovery and application of targeted drugs.

Here, we summarize the compounds and gene modification interventions recently associated with mitochondrial-dependent cell death. Pectolinarigenin^153^ and cinobufagin,^154^ isolated from an active ingredient in medicinal plants, are novel effective antitumor drug candidates for NPC and mainly inhibit tumor growth by activating mitochondrial-related apoptosis and accumulation of ROS. Bufalin (C_24_H_34_O_4_), a cardiotonic steroid, has been shown to inhibit cell proliferation, decrease the number of total viable cells, and induce G2/M phase arrest and apoptosis in NPC-TW 076 cells through ROS-induced endoplasmic reticulum (ER) stress and TRAIL pathways.^155^ Celastrol is an effective antioxidant and anti-inflammatory natural compound that inhibits the activity of NPC cells, causes G2/M cycle arrest, and induces mitochondria-related apoptosis.^156^ Another compound, grape seed proanthocyanidin, has a similar effect in NPC.^157^ The plant extract, acetogenin, is characterized by a single tetrahydrofuran (THF), which causes G2/M phase arrest, mitochondrial damage and apoptosis, and increases cytosolic and mitochondrial Ca^2+^ levels in NPCs.^158^ This antitumor effect is similar to that of the natural product, tetrandrine (TET).^159^ An interesting study focusing on an essential trace element showed that Na_2_SeO_3_ causes cell cycle arrest and affects the mitochondrial-mediated intrinsic caspase pathway with significant anti-proliferative and apoptosis-inducing effects in CNE-2 NPC cells, suggesting that Na_2_SeO_3_ might have therapeutic potential in the treatment of NPC.^160^ Additionally, human nuclear factor with BRCT domain protein 1 (NFBD1), an amplifier of the p53 pathway and also known as a mediator of DNA damage checkpoint protein (MDC1), is vital for protecting cells from apoptosis in NPC cells mediated through the p53-ROS-mitochondrial pathway.^161^ Liver cancer-1 (DLC-1) is lost at high frequency, mainly due to aberrant promoter methylation, and its tumor suppressor roles are not just limited to liver cancer cells as confirmed in multiple cancers.^162^ In NPC cells, targeting DLC-1 induces mitochondrial-mediated apoptosis and EMT arrest through the EGFR/AKT/NF-κB signaling pathway.^163^ Generally, the mobility group box 1 (HMGB1) protein is involved in a variety of biological processes and plays an important role in inflammation, immunity and tumor development.^164^ One research study revealed that HMGB1 is overexpressed in the HONE-1 NPC cell line and depression of HMGB1 leads to mitochondrial-mediated apoptosis by inhibiting the HMGB1/RAGE pathways^165^ (Table 4).

**Table 4:** Targeted mitochondria-related signaling in NPC

Targeting molecules	Agents or regulators	Effects	Reference
*Mitochondrial dynamics*			
AMPKɑ/Drp1	Metformin	Decreases mitochondrial fission and increases chemotherapy sensitivity to cisplatin	^101^
CyclinB1-Cdk1/Drp1	Cucurbitancin E	Decreases mitochondrial fission and increases chemotherapy sensitivity to cisplatin	^101^
COX-2/Drp1	Resveratrol	Inhibits mitochondrial fission and 5-Fu-resistance	^146^
TRIAP1	Knock down	Enhances mitochondrial fission and apoptosis	^143^
CDH1, RAC1, CDC42	GXHSWAQ-1	Enhances radiosensitisation by altering the mitochondrial structure	^144^
*Mitochondrial-related cell death*			
Bcl-2	Pectolinarigenin	Inhibits tumor growth by activating mitochondrial apoptosis and accumulation of ROS	^153^
Bcl-2	Cinobufagin	Blocks cells at the S phase, increases intracellular ROS levels, and induces mitochondrial apoptosis pathway	^154^
TRAIL	Bufalin	Induces G2/M phase arrest and mitochondrial cell death	^155^
MAPK	Celastrol	Triggers G1 and G2/M phase cell cycle arrest and Fas-mediated apoptosis	^156^
Bax/Bcl-2	GSPs	Induces apoptosis through the mitochondrial pathway and ultimately reduces cell viability	^157^
c-Jun/ERK	THF-ACGs	Inducers of the ESR that block cell proliferation	^158^
Bax, Bcl-xL, Bcl-2	Tetrandrine	Causes G0/G1phase arrest; increases ROS and Ca^2+^ production and apoptotic cell death	^159^
Bax, Bak, Bcl-XL	Na_2_SeO_3_	Inhibits cell proliferation and induces apoptosis	^160^
NFBD1/MDC1	Knock down	Induces apoptosis through the p53/ROS/mitochondrial pathway	^161^
DLC-1	Knock down	Leads mitochondrial apoptosis by targeting EGFR/AKT/NF-κB	^163^
HMGB1	Knock down	Decreases mitochondrial fission and increases chemotherapy sensitivity to cisplatin	^165^

Mitochondria play an important role in the pathogenesis of tumors by affecting mitochondrial DNA, dynamics, and cell death. The disruption of normal mitochondrial physiology is a hallmark of tumor development. Importantly, EBV- encoded proteins modulate mitochondrial function and thus alter bioenergetics and retrograde signaling pathways to promote tumorigenesis. Therefore, identification of effective mitochondrial molecules and signaling pathways in EBV-associated tumor is essential for developing novel therapeutic strategies.

## EBV and the ubiquitin-proteasome system (Ups)

Post-translational modification of proteins by ubiquitin (Ub) is a key regulatory event in various cellular activities. Ubiquitination is a dynamic and reversible process, wherein ubiquitin chains are cleaved by deubiquitinase (DUB).^166^ Ubiquitin signaling comprises three major components of the ubiquitination process known as ubiquitin-activating enzyme (E1), ubiquitin-conjugating enzyme (E2), and ubiquitin ligase (E3). E1 activates and transfers ubiquitin to the E2 enzyme and then E2 interacts with the E3 ligase. The E3 ligase binds and transfers ubiquitin from the E2 enzyme to target-specific proteins for degradation. The deregulation of the ubiquitin system leads to human pathogeneses, especially cancer. Alterations in the ubiquitin system are known to occur during the initiation and progression of cancer, and this knowledge is starting to be exploited for the development of novel strategies to treat cancer.^167^

DUBs maintain ubiquitin system homeostasis by cleaving polyubiquitin chains or completely removing ubiquitin chains from ubiquitinated proteins and then generating and recycling free ubiquitin. Deubiquitination also has an important function in regulating ubiquitin-dependent pathways, including cell cycle regulation, cell death, protein degradation, protein function, gene expression, and signal transduction.^168,169^ Imbalances in DUB activities are involved in multiple diseases, including cancer, inflammation, neurological disorders, and microbial infections. DUB inhibitors (DIs) are becoming potential therapeutic approaches against cancer.^170^ Most DIs are small-molecular compounds, exerting their function by suppressing DUB activity. Thus far, about 100 DUBs have been identified and classified into two categories, including cysteine proteases and metalloproteases. The cysteine proteases comprise ubiquitin-specific protease (USP), ubiquitin C-terminal hydrolase (UCH), Machado-Josephin domain protease (MJD), ovarian tumor protease (OTU), and motif interacting with Ub-containing novel DUB family (MINDY). The metalloproteases comprise Jab1/Mov34/Mpr1 Pad1 N-terminal (MPN) (JAMM) domain protease.^171,172^

### EBV-encoded proteins and ubiquitin system

All viruses need host machinery to maintain infection and replication. UPS is involved in all aspects of cellular activity. Oncoviruses rely on this system at many levels and even hijack the ubiquitin system to meet their survival needs. The same is also true for EBV. EBV nuclear antigen 1 (EBNA1) plays important roles in EBV latent gene expression. EBNA1 competitively interacts with the p53 binding site on ubiquitin carboxyl-terminal hydrolase 7 (USP7), which is also known as herpes virus-associated ubiquitin-specific protease (HAUSP). The EBNA1 and USP7 interaction can promote cell survival and contribute to EBNA1 functions at the EBV oriP and inhibit p53-mediated antiviral responses.^173^ EBV-encoded latent membrane protein 1 (LMP1) can induce the expression of ubiquitin carboxyl-terminal hydrolase isozyme L1 (UCHL1) and it may contribute to viral transformation and the progression of lymphoid malignancies.^174^ EBV early-lytic phase gene BRLF1 encoded Rta, which initial viral gene transcription. Rta can be ubiquitinated by E3 ubiquitin ligase TRIM5a. Overexpression of TRIM5a reduces the transactivating capabilities of Rta, whereas reducing TRIM5a expression enhances EBV lytic protein levels and DNA replication.^175^

Besides utilizing host DUBs, EBV can also encode the viral deubiquitinating enzyme (v-DUB), BPLF1, which is an immune evasion gene product that can suppress antiviral immune responses during primary infection. BPLF1 is expressed during the late phase of lytic EBV infection and is incorporated into viral particles. It can eliminate K63- and/or K48-linked ubiquitin chains and act as an active DUB during the productive lytic cycle and EBV infection.^176^

### Disturbing of ubiquitin-proteasome system in EBV-related cancer

#### Cell cycle

Cell cycle regulation is one of the hallmarks of virus-mediated oncogenesis. EBV-encoded LMP1 is an important tumorigenic protein. Unlike many other cancers, p53 is highly expressed in NPC. LMP1 enhances p53 accumulation through two distinct ubiquitin modifications. LMP1 promotes p53 stability by suppressing K48-linked ubiquitination of p53 by E3 ligase murine double minute 2 (MDM2). LMP1 inhibits MDM2-mediated p53 ubiquitination associated with p53 Ser20 phosphorylation and also induces K63-linked ubiquitination of p53 by interacting with TRAF2, thus disrupting tumor cell apoptosis and cell cycle arrest.^177^ Another essential EBV latent protein 3C (EBNA3C) stabilizes Cyclin D2 to regulate cell cycle progression by directly binding to Cyclin D2, and co-localizing together in nuclear compartments.^178^ EBNA3C interacts with Bcl6 and facilitates its degradation through the ubiquitin-proteasome-dependent pathway, and suppresses Bcl6 mRNA expression by inhibiting the transcriptional activity of its promoter. EBNA3C-mediated Bcl6 regulation significantly promotes cell proliferation and cell cycle by targeting Bcl2 and CCND1.

During viral lytic replication, EBV protein kinase (PK) phosphorylates p27Kip1 so that it is ubiquitinated by the Skp1/Cullin/F-box protein (SCF^Skp2^) ubiquitin ligase and degraded in a proteasome-dependent manner. Unlike the cyclin E-CDK2 activity, the EBV PK activity is not inhibited by p27Kip1. Overall, EBV possesses its own strategy to degrade p27Kip1 upon onset of productive replication, contributing to provision of an S-phase-like cellular environment with high CDK activity.^179^

#### Cell death

Cell death is a critical component of the host immune response against invading microbial pathogens. As is discussed above, EBV-LMP1 disrupting tumor cell apoptosis through p53. EBV is also capable of preventing necroptosis. Necroptosis is a relatively newly discovered pathway of programmed cell death, the deregulation of which is related to various inflammatory diseases and cancer.^180^ LMP1 is armed with mechanisms independent of the RIP homotypic interaction motif (RHIM)-domain interactions to block necroptosis. First, LMP1 is able to interact with both receptor-interacting protein kinase 1 (RIPK1) and receptor-interacting protein kinase 3 (RIPK3) by C-terminal activation region 2 (CTAR2), which is necessary for this interaction. Breaking the combination of RIPK1 and RIPK3 inhibits the formation of necrosomes. Second, and more importantly, the ubiquitination of the two receptor-interacting proteins can be modulated by LMP1. LMP1-mediated promotion of K63-polyubiquitinated RIPK1, suppression of RIPK1 protein expression, and inhibition of K63-polyubiquitinated RIPK3 induces a switch in cell fate from necroptotic death to survival.^181^

#### DNA damage

DNA damage signaling and repair pathways are important determinants of both cancer development and cancer therapy. EBV nuclear protein expressed in gastric carcinomas (GC), EBNA1, focusing on promyelocytic leukemia (PML) nuclear bodies (NBs), which play important roles in apoptosis, p53 activation. EBV infection of GC cells leads to degradation of PML NBs through the action of EBNA1 interacting with USP7, resulting in impaired responses to DNA damage and promotion of cell survival. Therefore, PML disruption by EBNA1 is one mechanism by which EBV may contribute to the development of gastric cancer.^182^ O^6^-methylguanine-DNA methyltransferase (MGMT) is a key DNA repair enzyme contributing to the chemoresistance, which protects NPC cells from DNA damage by enhancing the capacity for DNA repair.^183^ Research findings showed that ubiquitin-conjugating enzyme E2 B (UBE2B) collaborates with E3 ubiquitin ligase RAD18 to mediated MGMT ubiquitination and degradation.^184^ Rad18 is also responsible for monoubiquitination of PCNA, which initiates several cellular DNA repair processes. V-DUB BPLF1 targets ubiquitinated PCNA and disrupts trans-lesion synthesis (TLS). Deubiquitination of PCNA by the viral DUB can disrupt repair of DNA damage by compromising recruitment of TLS polymerase to stalled replication forks.^185^ BPLF1 interacts directly with Rad18, and overexpression of BPLF1 results in increased levels of the Rad18 protein, suggesting that it stabilizes Rad18. Next, expression of functionally active BPLF1 causes relocalization of Rad18 into nuclear foci, which is consistent with sites of cellular DNA replication that occur during S phase, which contributes to virus replication and infectivity.^186^ Transcription factor p53 is a critical mediator that is directly phosphorylated and stabilized by DNA damage signaling kinases, which triggers the apoptotic program.^187^ Zhang et al.^188^ found that RING-finger domain-containing ubiquitin E3 ligase tripartite motif–containing 21 (TRIM21) repressed p53 expression by mediating guanine monophosphate synthase (GMPS) ubiquitination and degradation. TRIM21 protects NPC cells from radiation-induced apoptosis by manipulating the GMPS–p53 cascade.^188^ In addition, TRIM21 is also responsible for the polyubiquitination of prohibitin 1 (PHB1), a pleiotropic protein that functions as a tumor suppressor. Ubiquitination and degradation of PHB1 leads to transcriptional activation of NF-κB and STAT3.^189^ TRIM21 mediates small G-protein signaling modulator 1 (SGSM1) ubiquitination degradation and inhibits the MAPK pathway activation.^190^ Deubiquinase ubiquitin carboxyl-terminal hydrolase L1 (UCHL1) from the UCH family forms complexes with p53/MDM2/ARF and deubiquitinates p53 promoting p53 signaling, which is involved in NPC pathogenesis.^191^

#### Akt signaling

**Fig. 4:**
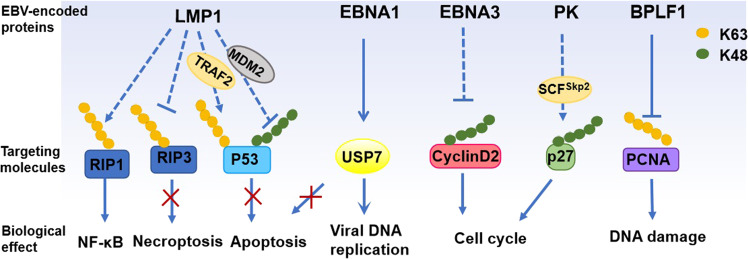
EBV-encoded proteins modulate host ubiquitin signaling pathways. EBV regulates degradation and non-degradation ubiquitination of key molecules in cell signaling pathways through hijacking host ubiquitinase and deubiquitinase, or directly encoding viral deubiquitinase. The ubiquitinases in orange cycle represents activated by EBV protein, and the gray represents for suppressed ubiquitinase

Deregulation of protein kinase B (Akt) signaling plays a vital role in the regulation of tumorigenesis in human cancers.^192^ K63-linked ubiquitination of Akt is required for Akt membrane translocation and activation.^193^ E3 ligase S-phase kinase-associated protein 2 (Skp2) overexpression is correlated with poor prognosis in NPC patients, Skp2-mediated ubiquitination and mitochondrial localization of Akt drive tumor growth and resistance to cisplatin.^194,195^ TNF-receptor-associated factor (TRAF) proteins are key adaptor molecules containing E3 ubiquitin ligase activity, which plays a critical role in multiple cell signaling pathways. TRAF6 binds to and ubiquitinates Krüppel-like factor 4 (KLF4), a complex transcription factor that acts as a tumor-promoting gene in NPC. This leads to KLF4 K32/K63-linked ubiquitination and stabilization, which results in NPC^196^ (Fig. 4).

### The natural compound, neoalbaconol, targets the ubiquitin system in EBV-related cancer

The ubiquitin-proteasome system (UPS) is emerging as a novel target for anticancer drugs and molecular diagnostics.^197^ Escape from programmed cell death plays a critical role in cancer. Therefore, investigating the role of necroptosis in cancer has been of high interest. We found that necroptosis is one of the major cell death forms induced by neoalbaconol. Natural compounds derived from plants, fungi, and marine organisms are widely used as anticancer drugs in preclinical/clinical development or in clinic.^105^ Neoalbaconol down-regulates E3 ubiquitin ligases cellular inhibitors of apoptosis protein 1/2 (cIAP1/2) and TRAFs to abolish K63-linked ubiquitination on RIPK1.^198^ This induces the activation of the non-canonical NF-κB pathway and increases the transcription activation of TNFα. Moreover, neoalbaconol induces RIPK3-mediated reactive oxygen species (ROS) production that participates in necroptosis.^198^ RIPK1/NF-κB-dependent TNFα secretion and RIPK3-dependent ROS generation contribute to neoalbaconol-induced necroptosis.

Ubiquitinases and deubiquitinases are emerging as potential therapeutic targets in many diseases, especially cancer. In this chapter, we summarize the involvement of these enzymes in cancer signaling and cell regulation and illustrate the potential role of the ubiquitin system in response to cancer therapeutics (Table 5). We also discussed how the onco-virus EBV fights with the host by utilizing host ubiquitin signaling or even encoding v-DUB to hijack host cellular processes. Deciphering the various functions of ubiquitinases and deubiquitinases will be challenging but should help provide a greater understanding of how its deregulation results in tumorigenesis and development. As for the potential therapeutic targets, the ultimate challenge is to translate these basic research findings into novel and more effective cancer therapies.

**Table 5:** Ubiquitinase in EBV-related tumorigenesis

Type	Enzyme	Target	Function	Reference
Ubiquitin E3 ligase	UBE2B	MGMT	Chemoresistance	^184^
	RAD18	MGMT	Chemoresistance	^184^
	TRIM21	GMPS	Apoptosis inhibition	^188^
		PHB1	Cell proliferation	^189^
		SGSM1	MAPK pathway activation	^190^
	Skp2	Akt	Chemoresistance and cell proliferation	^193^
	TRAF6	KLF4	Tumorigenesis	^196^
	TRAF2	P53	Disrupting tumor cell apoptosis	^177^
	TRIM5a	Rta	Inhibit EBV lytic replication	^175^
Deubiquintylase	UCHL1	p53	Tumorigenesis	^191^
	USP7	p53	Inhibit antiviral responses	^173^
v-Deubiquintylase	BPLF1	PCNA	Disrupt repair of DNA damage	^185^

## Oxidative stress in EBV-associated diseases

The regulation of redox homeostasis is fundamental to maintaining normal cellular functions and ensuring cell survival. Cancer cells are characterized by increased aerobic glycolysis and high levels of oxidative stress.^199^ This oxidative stress is exerted by reactive oxygen species (ROS) that accumulate as a result of an imbalance between ROS generation and elimination.^200^ ROS are constantly produced by both enzymatic and non-enzymatic reactions.^200^ Enzyme-catalyzed reactions that generate ROS include those involving NADPH oxidase, xanthine oxidase, uncoupled endothelial nitric oxide synthase (eNOS), arachidonic acid and metabolic enzymes such as the cytochrome P450 enzymes, lipoxygenase and cyclooxygenase. The mitochondrial respiratory chain is a non-enzymatic source of ROS.^200^ The produced ROS include superoxide, hydrogen peroxide, singlet oxygen and reactive nitrogen species. Usually, determination of cellular redox status by a balance between levels of ROS inducers and ROS scavengers. The production of reactive oxygen species (ROS) can be induced by hypoxia, metabolic defects, endoplasmic reticulum (ER) stress and oncogenes. Conversely, ROS are eliminated by the activation of the transcription factor nuclear factor erythroid 2-related factor 2 (NRF2), the production of glutathione and NADPH, the activity of tumor suppressors (such as breast cancer susceptibility 1 (BRCA1), p53, phosphatase and tensin homolog (PTEN) and ataxia telangiectasia mutated (ATM)) and the action of dietary antioxidants.^200^ Nowadays, many compounds have been described as antioxidants. These include compounds with free sulfhydryl groups, including N-acetylcysteine (NAC) and lipoic acid, compounds with multiple double bonds and conjugation (e.g., carotenoids, tocopherols and retinoids, among others) and polyphenols (e.g., epicatechingallate and quercetin, among others). Compounds that inhibit reactive oxygen generation (i.e., NADPH oxidase inhibitors), xanthine oxidase inhibitors and compounds that induce oxidant defenses (e.g., Nrf2 activation-sulforaphane, among others) have also been allocated to this category.^201^

ROS-mediated oxidative stress has an increasingly well-recognized broad function in oncogenesis.^202^ Generating oxidative stress is a critical mechanism by which host cells defend against infection by pathogenic microorganisms.^203^ Several reports have shown that both DNA and RNA viruses can lead to oxidative stress by encoding various products and play roles in virus survival. For example, HBV encoded HBx^204^ and HSV-1 encoded UL12.5^205^ can induce oxidative stress by degradation of mtDNA; KSHV encoded Rac-1^206^ and HIV encoded Gp120, Tet or Nef^207^ can induce oxidative stress by regulating the NOX pathway.

EBV, a member of γ-herpesviruses, is an important cancer causing virus.^4^ NPC is an infection-associated cancer strongly driven by EBV.^11,208^ So far, several evidences showed increased levels of ROS in cells and in NPC patients. In vitro, EBV infection can induce an increase in malondialdehyde (MDA) levels and decreases in catalase and superoxide dismutase (SOD) activities. Additionally, a positive correlation was observed between *BZLF1* and antioxidant enzyme genes expression, which also confirmed a role of EBV lytic reactivation in oxidative stress.^209^ Notably, reactive oxygen signaling can distinguish EBV-positive versus EBV-negative tumors based on the finding that elevated levels of ROS are observed in EBV-positive tumors but not in EBV-negative tumors.^210^ On the other hand, oxidative stress inducers, such as H_2_O_2_ or FeSO_4_, can cause EBV lytic cycle induction as demonstrated by BZLF1 gene expression.^211^ Moreover, various drugs that induce or inhibit EBV lytic reactivation also act by generating oxidative stress.^212,213^ Thus, a vicious cycle could be initiated whereby reactivation of EBV and ROS production amplify each another. In vivo, overexpression of iNOS and the accumulation of 8-OHdG, critical biomarkers of oxidative stress, were found in NPC patients.^214,215^ Huang et al.^216^ detected the serum 8-OHdG using ELISA in NPC patients and found that all cases of NPC were positive for 8-OHdG and therefore, 8-OHdG is proposed as a potential biomarker for evaluating the risk of NPC. Su et al.^217^ observed decreased SOD activity in NPC that is consistently associated with EBV. These observations indicate that NPC biopsies present a high level of oxidative stress.

Moreover, other EBV-associated tumors also present high levels of oxidative stress. For instance, Cerimele et al.^210^ demonstrated that EBV-positive BL expresses high levels of activated mitogen-activated protein kinase and reactive oxygen species (ROS), and that ROS directly regulate NF-κB activation. In EBV-associated gastric cancers, Kim et al.^218^ reported EBV induces high levels of oxidative stress and thus regulates cell viability.

Additionally, it is reported that several EBV-encoded products, including EBNA1, LMP1, and EBER are associated with oxidative stress. Possible mechanisms by which EBNA1 could induce ROS, include increasing levels of oxidases (e.g., NOX1 and NOX2) or affecting the levels of antioxidant enzymes, such as SOD1, peroxiredoxin 1, peroxiredoxin 6, or glutathione S-transferase.^16,219,220^ The mechanism of inducing oxidative stress by LMP1 was via activating of NOX2 and Nrf2 pathway (unpublished data). EBER that induces interleukin (IL)-10 and IL-10 has been implicated in the activation of mitochondrial ROS.^210^ Based on the reported preclinical and clinical evidence, it is indicated the oxidative stress is involved in EBV infection.

Bonner et al.^201^ has classified tumors by signaling pathways and proposed a concept of ROS-driven tumors. So far, several types of tumors have been classified into reactive oxygen species (ROS)-driven tumors and include EBV-associated Burkitt’s lymphoma, Hodgkin’s disease, gastric carcinoma, hepatitis C virus-related hepatocellular carcinoma, ultraviolet A-induced melanoma, inflammatory bowel disease-induced colon carcinoma, schistosomiasis-induced bladder cancer, tobacco-induced oral and lung carcinoma, and epidermolysis bullosa-associated squamous cell carcinoma. These cancers are characterized by activation of high expression levels of NF-κB, Akt and wild-type p53 and PTEN.^201^ According to the signaling events in the reactive oxygen-driven tumor and the fact of high oxidative stress in NPC, it is proposed that NPC is a reactive oxygen-driven tumor.

Regarding to the important role of ROS in EBV-associated diseases, the monitoring and detection of ROS is vital important to the therapy of EBV-related malignancies. So far, there are several ways to detect the level of intracellular oxidative stress. (a) Total ROS level (combined with ROS dye by flow cytometry); (b) mitochondria ROS level (using flow cytometry combined with mitochondrial-specific ROS dye MitoSox red); (c) intracellular NADP^+^/NADPH ratio (NADP^+^/NADPH kit); (d) intracellular GSSG/GSH ratio (GSSG/GSH kit); (e) Expression and activity of oxidoreductase. For the detection of oxidative stress level of tumor patients, pathological samples and serum samples can be collected, which can be reflected by detecting the content of oxidative stress marker 8-hydroxydeoxyguanosine (8-OHdG). 8-OHdG is one of the most common DNA damage products in response to oxidative stress, and has been recognized as a reliable and stable biomarker of oxidative stress.^221,222^ The level of 8-OHdG in tissue samples is positively correlated with the degree of oxidative stress, so the level of 8-OHdG in blood or pathological tissue samples can be detected by ELISA or immunohistochemistry to reflect the degree of oxidative stress.

Scavenging ROS might be a good way for NPC prevention and therapy. In our group, we demonstrated that scavenging ROS by NAC can decrease the level of ROS and increase the sensitivity of NPC to radiation (unpublished data). In addition, natural compounds, normally found in foods, are gaining increased attention due to their proposed cancer preventive and therapeutic activities and potential greater safety compared to synthetic compounds.^223^ In therapy of EBV-associated cancers, curcumin, resveratrol and EGCG reportedly show great potential.^224–226^

## EBV lytic reactivation in EBV-associated diseases

**Fig. 5:**
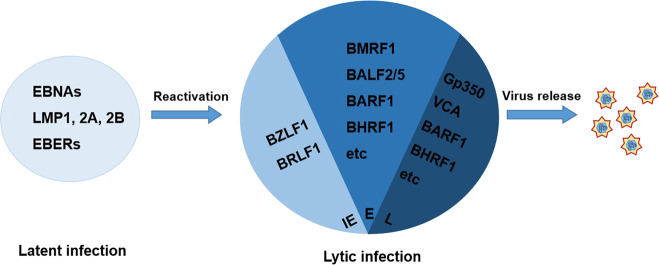
EBV encodes different products in latent and lytic infection. During latency, a limited number of viral products, such as EBNAs, LMP1, LMP2A, LMP1 2B, EBERs are expressed and the viral genome exists in the nucleus as an episome. Upon reactivation, EBV briefly passes through three consecutive lytic phases, including immediate early (IE), early (E), and late (L). The viral IE genes BZLF1 and BRLF1 are first transcribed to encode the transactivators, Zta and Rta, respectively, followed by expression of the early genes required for EBV genome replication. After EBV DNA replication, late genes are expressed that encode mainly viral structural proteins, including capsid antigens and membrane proteins, followed by viral genome encapsulation and the production of mature virions

Like all herpesviruses, EBV establishes a latent infection that is periodically reactivated into the productive lytic cycle.^227^ As showed in Fig. 5, EBV-encoded different products in latency and lytic phase. Following reactivation, the lytic genes of EBV are expressed in a temporally regulated manner, including immediate early (IE), early (E) and late (L). The latent-lytic switch is tightly controlled by both cellular and viral factors.^227^ About the history of EBV lytic research, there are several major milestones. In 1985, VCA-IgA levels detected in mass serological screening of NPC patients in China, confirming NPC risk.^21^ Countryman et al.^228^ demonstrated that BZLF1 is the switch from latent to lytic cycle. In 1987, EBV DNA was detected in HL biopsies.^228^ In 1989, Q-PCR was used to detect cell-free EBV DNA as NPC biomarker.^23^ The latest reports showed an important transcription factor MYC can also control the EBV lytic switch.^229^ EBV ncRNA from an NPC induces an inflammatory response that promotes virus production.^230^

### EBV lytic cycle and tumorigenesis

In recent years, evidence of a contribution of the lytic cycle to EBV-induced oncogenesis has emerged and has become a hot topic in this field.^4,65,231^ EBV lytic phase is associated with NPC, GC, and multiple lymphomas, including Burkitt lymphoma, Hodgkin lymphoma et al. and confers therapeutic resistance to tumor cells.^4^ In our lab, we also indicated that the EBV spontaneous lytic infection exists in NPC and lymphoma cells.^36^ Notably, in NPC, the expression of lytic EBV genes was observed in the upper layers of differentiated epithelium but not in the undifferentiated basal layers.^10,34^ Interestingly, Christian analyzed the degree of EBV lytic in EBV-related diseases. The sequence of lytic replication is: oral hairy leukoplakia > infectious mononucleosis > primary effusion lymphoma > nasopharyngeal carcinoma/gastric carcinoma > diffuse large B-cell lymphoma/Burkitt’s lymphoma/post-transplant lymphoproliferative disease/NK-cell or T-cell lymphoma > Hodgkin’s lymphoma.^231^

In addition, EBV lytic reactivation is also related to several other diseases, including multiple sclerosis (MS), systemic lupus erythematosus (SLE), rheumatoid arthritis (RA) and Sjogren’s syndrome (SS). MS is a chronic and disabling inflammatory and neurodegenerative disease of the central nervous system that affects 350,000 people in the USA and ~2 million worldwide. It is reported that MS caused an increased anti-EBV antibodies titer against multiple antigens and MS risk was up to 30-fold higher in individuals with the highest titers of anti-EBV antibodies as compared to those with the lowest.^6,232^ SLE is a multisystem autoimmune disease with varied presentations, progression, and symptoms experienced by patients. Patients with SLE have high frequencies of EBV-infected cells, higher plasma viral loads and higher early antigen IgG levels, which suggest active EBV lytic replication in SLE patients and serological measures of EBV reactivation may provide additional biomarkers to identify individuals at higher risk transitioning to clinical SLE.^6,233^ RA is a common autoimmune disease with a prevalence of ~1% and an incidence of 5–50 per 100,000 per year with three times more female than male patients. Common symptoms include arthritis, cardiovascular complications, metabolic syndrome, cognitive dysfunction, and depression. Presence of EBV DNA/RNA has been demonstrated in PBMCs, saliva, synovial fluid, and synovial membranes of RA patients. Furthermore, 10-fold higher frequencies of EBV-infected B-cells have been observed in RA patients compared to healthy controls. Interestingly, EBV DNA was found in many of the plasma cells producing cyclic citrullinated peptide (CCP) antibodies localized in synovial tissues of RA patients. These results indicate a widespread lytic EBV infection in RA patients.^61,234^ SS is a rather common autoimmune disease with a prevalence of about 0.5% and an incidence of 3–6 per 100,000 per year with a female preponderance (nine out of 10 SS patients are women). Increased viral load and EBV proteins have been found in salivary and lacrimal glands of SS patients indicating active infection, and elevated levels of EBV-directed antibodies have been found in the circulation.^235^ The above evidence demonstrates that the MS, SLE, RA, and SS patients have active EBV lytic replication. Elimination of the infection or reduction of the viral load by reducing the antigenic stimulation could reduce titers of autoantibodies and possibly have a clinical benefit.

Research findings have documented that EBV lytic reactivation is a major risk factor for the development of EBV-related diseases. They mainly contribute to the induction of genome instability, the counteraction of host immune responses, the resistance of cell death and the promotion of tumor development, progression and invasiveness.^236,237^ The EBV lytic proteins and tumorigenic functions are listed in Table 6. Additionally, induced activation of EBV contributes to carcinogenesis. It has been reported several lifestyle factors can induce transformation of EBV from the latent to the lytic stage and contribute to NPC occurrence, such as salted fish or preserved food.^238^ Besides, cigarette smoking can induce EBV lytic reactivation and increase the risk of NPC.^239^ Moreover, several cellular stresses were also reported to contribute to EBV reactivation and lead to an increase of NPC risk, such as oxidative stress, hypoxia and inflammation.^237^ In addition, lytic EBV replication might condition the tumor microenvironment for EBV-associated malignancies through attraction of monocytes via CCL5 and their differentiation into immune-suppressive tumor-associated macrophages (TAMs).^231^ The above-mentioned function of EBV lytic reactivation indicated that the lytic cycle plays an important role in the pathogenesis of EBV-related diseases.

**Table 6:** EBV lytic proteins and tumorigenic functions

Lytic protein	Lytic phase	Tumorigenic function
Zta	IE	Induction of IL-6, IL-8, IL-10, IL-13, and VEGF secretion
Rta	IE	Induction of genomic instability and progressive malignancy
BHRF1	E	Anti-apoptosis
BARF1	E	Anti-apoptosis
BCRF1	E	Anti-apoptosis, homologous to IL-10
BALF3	E	Induction of genomic instability
BGLF4	E	Induction of chromosomal abnormality and DNA damage
BGLF5	E	Induction of genomic instability
DNase	E	Induction of genomic instability
BNLF2a	E	Immune evasion (inhibits the transporter of antigen processing)
BILF1	E	Immune evasion (induces major histocompatibility complex (MHC) class I internalization and degradation)
BGLF5	E	Immune evasion (mediates host shut‑off)
BNRF1	L	Induction of centrosome amplification and chromosomal instability

Currently, detection of EBV based on EBV lytic reactivation might be one of the most important approaches for the prevention and personalized treatment of EBV-associated cancers. For example, Rta-IgG, EAD-IgA, VCA-IgA, etc. are antibodies produced based on products of EBV lytic activation.^4,240^ In addition, in areas with high incidence of NPC, detection of EBNA1-IgA and VCA-IgA by ELISA has become a routine screening method for high-risk groups of NPC, and has been incorporated into the NPC screening standard of the National Health and Family Planning Commission.^241^ In addition, accumulating research data supports the use of plasma or serum EBV DNA as a tumor marker.^242–246^ Plasma or serum EBV DNA is gradually being adopted in clinical applications and has been shown to be correlated with tumor burden, TNM stage, response to therapy, and survival in NPC patients. Most notably, two phase 3 trials are under way, in which patients are randomly assigned to adjuvant regimens of differing intensities depending on whether EBV DNA is detectable after chemoradiotherapy (NCT02135042 and NCT00370890, Clinical Trails.gov).^208^ In addition, NP brush/swab sampling combined with EBV DNA detection has been a valuable supplement in the diagnosis of NPC because of its high sensitivity and specificity.^247^

EBV DNA detection also has a board application in other EBV-associated diseases except NPC. (1) For IM patients, the detection of EBV DNA may be helpful to cases in which EBV antibody detection results are difficult to explain. (2) For immunodeficiency patients related to EBV infection, such as XLPD and PTLD, duo to the insufficient of the antibody in immunodeficiency patients, the detection of EBV DNA is helpful for the diagnosis of primary EBV infection, and whole blood or PBMC specimens is recommended for EBV detection. (3) CAEBV (Chronic active EBV infection) is a serious EBV infection disease, and the EBV load in peripheral blood of CAEBV patients is significantly higher than that of healthy carriers. The EBV DNA in serum or plasma of patients with CAEBV was positive, or the EBV DNA in PBMC of peripheral blood was higher than 10^2.5 copies/μg DNA. So, the detection of EBV DNA is a good method for CAEBV. (4) For lymphoma patients, serum/plasma EBV DNA can be used as a marker of tumor burden in patients with EBV-related lymphoma, and may be used for treatment effect evaluation and prognosis judgment.

### Antiviral agents targeting EBV lytic cycle

EBV infection has been shown to significantly contribute to carcinogenesis. Thus, Screening for anti-EBV agents have been developed as novel strategies for treatment of EBV-associated malignancies. So far, most of anti-EBV agents were targeting the proteins expressed in EBV lytic cycle. Valpromide (VPM), an amide derivative of valproic acid (VPA), has been proven to prevented expression of two EBV genes, BZLF1 and BRLF1, which mediate lytic reactivation. Unlike VPA, VPM did not activate cellular gene expression. VPM will be useful in probing the mechanism of EBV lytic reactivation and may have therapeutic application.^248,249^ Verma et al.^250^ have identified that Spironolactone, a mineralocorticoid receptor antagonist approved for clinical use, inhibits infectious EBV production by inhibiting EBV SM protein function. SM protein is a regulatory protein early in lytic replication of EBV, which is essential for efficient gene expression and infectious virion production. Maribavir (MBV), which is newly approved drug for use against human cytomegalovirus (HCMV) infection in allogeneic stem cell and bone-marrow transplant recipients, is of special interest because it is also a potent inhibitor of EBV replication.^251^ MBV on EBV lytic replication is produced mainly by inhibiting the enzymatic activity of EBV-PK and thus the viral DNA replication, as well as through suppression of EBV lytic gene expression.^252,253^ It’s worth noting that DNAzyme (DZ1) that was engineered to specifically target the LMP1 mRNA, which is a latent oncoprotein of EBV.^254^ As the research progresses, increasing evidence indicate that the high expression of LMP1 during EBV lytic cycle can promote the viral lytic replication. In our previous research, we have found that EBV DNA copy number was significantly decreased in DZ1-treated patients.^255^ Which consisted the critical role of LMP1 in EBV lytic reactivation. These suggesting that lytic inhibition may be an important mechanism for DZ1 against EBV-related cancer.

Currently, a wide variety of active phytochemicals or dietary ingredients have been identified as inhibitors of the EBV lytic cycle^256–263^ (Table 7). Most of the natural compounds act as antiviral agents by inhibiting lytic proteins. Our previous studies have shown that EGCG could effectively inhibit the constitutive lytic infection of EBV at the DNA, gene transcription and protein levels.^264^ And the molecular mechanism underlying EGCG inhibition of EBV lytic infection involves downregulation of LMP1.^265^ Through inhibiting the EBV lytic cycle, anti-EBV agents may decrease viral particle production, block the transmission of the virus from cell to cell and are, therefore, valuable in chemoprevention or clinical treatment of EBV-associated malignancies. Although these were still in vitro studies. These findings pave the way for development of antiviral drugs with new mechanisms of action directed against EBV in the future.

**Table 7:** Antiviral agents targeting EBV lytic cycle in EBV-related diseases

Agents	Mechanism	Model	Reference
Valproic acid	Inhibits Zta and Rta expression	In vitro (Burkitt’s lymphoma)	^248^
Valpromide	Inhibits Zta and Rta expression	In vitro (Burkitt’s lymphoma)	^249^
Maribavir	Inhibiting the enzymatic activity of EBV-PK	In vitro (Burkitt’s lymphoma)	^252,253^
Spironolactone	Inhibits SM expression	In vitro (Burkitt’s lymphoma, GC)	^250^
Dnazyme	Inhibits LMP1 expression	In vivo (NPC)	^254,255^
Epigallocatechin gallate	Inhibits EBV lytic gene expression	In vitro (Burkitt’s lymphoma, NPC)	^258,264,265^
Curcumin	Inhibits BZLF1 gene transcription	In vitro (Burkitt’s lymphoma)	^256^
Sulforaphane	Reduces the transactivation activity of the BRLF1 gene	In vitro (NPC)	^257^
Protoapigenone	Reduces the transcriptional function of Zta	In vitro (Burkitt’s lymphoma)	^259^
Moronic acid	Suppresses the transactivation function of Rta	In vitro (Burkitt’s lymphoma)	^260^
Emodin	Inhibits EBV IE protein expression and DNA replication	In vitro (Burkitt’s lymphoma)	^261^
Glycyrrhizic acid	Inhibits EBV IE protein expression and DNA replication	In vitro (Burkitt’s lymphoma)	^262^
Vitamin C	Reduces EBV EA IgG and VCA IgM antibody levels	In vivo (CAEBV)	^263^

Recently, studies have showed that Ephrin receptor A2 (EphA2) is critical for EBV entry into the epithelial cells. EphA2 interacted with EBV entry proteins gH/gL and gB to facilitate EBV internalization and fusion. And the interaction is mediated by the EphA2 extracellular domain (ECD).^266,267^ In addition, Peng et al.^268^ have identified that small-molecular inhibitor of EphA2, ALW (ALW-II-41–27), strongly inhibited the proliferation of gastric cancer in vitro and in gastric cancer patient–derived xenografts. These data indicated that specifically inhibition of EphA2 may be an effective strategy in gastric cancer therapy. Which also opens up potential possibilities for antiviral agents targeting of EphA2. The discovery of EphA2 as an EBV epithelial cell receptor has important implications for EBV pathogenesis and may uncover new potential targets that can be used for the development of novel intervention strategies.

## Non-coding RNA (ncRNA) and EBV-associated tumors

### The role of EBV-encoded ncRNA

EBV was the first human virus found to encode microRNAs (miRNAs) since 2004.^19^ The EBV latent non-coding RNAs include BamHI-A rightward transcript miRNAs (BART miRNAs), BHRF1 miRNAs and EBER1 and EBER2.^4^ EBV-encoded miRNAs are generated from two regions of the viral genome, within the apoptosis regulator *BHRF1* gene locus and near the BamHI A region in a latency type-dependent manner.^269^ EBV encodes 44 mature BART miRNAs, a number of which have been proven to promote carcinogenesis by targeting host genes or self-viral genes. BART miRNAs, which are encoded by the BamHI region of EBV, are reported to be abundant in NPC and have potential value in early diagnosis of NPC. Accumulating evidence suggests that EBV-miR-BARTs play a critical role in host cell survival, immune escape, cell proliferation, apoptosis, and cancer metabolism, promoting the generation of NPC.^270^

To understand the roles of EBV miRNAs in the pathogenesis of NPC, Chen et al.^271^ utilized deep sequencing technology to characterize the EBV miRNA transcriptome in clinical NPC tissues. Sequence analysis revealed that most of the highly abundant EBV miRNAs shared their seed sequences (nucleotides 2–7) with human miRNAs, suggesting that seed sequence content might be an important factor underlying the differential accumulation of EBV miRNAs. These observations not only provided a potential linkage between EBV miRNAs and human malignancy, but also suggested a highly coordinated mechanism through which EBV miRNAs might mimic or compete with human miRNAs to affect cellular functions. More importantly, EBV-encoded miRNAs compromise the attraction of CD8^+^ T cytotoxic lymphocytes into the tumor microenvironment by down-regulating CXCL11 expression and also inhibit antigen presentation on MHC class I molecules to these CD8^+^ T cells, rendering the microenvironment of EBV-associated malignancies immune-suppressive.^231^

#### EBV-miR-BART1–5p

EBV-encoded miR-BART1-5p detection by nasopharyngeal brush sampling could act as an efficient and less invasive method assisting clinical diagnosis of NPC (with 93.5% sensitivity and 100% specificity), and the miR-BART1-5p relative expression level in nasopharyngeal brush samples was reflective of NPC progression.^272^ EBV-miR-BART1-5p directly targeted the α1 catalytic subunit of AMP-activated protein kinase (AMPKα1) and consequently mediated the AMPK/mTOR/HIF-1 pathway, which promoted NPC cell anomalous aerobic glycolysis and angiogenesis, ultimately leading to uncontrolled growth of NPC.^273^ Also, EBV-miR-BART1 directly targeted the cellular tumor suppressor PTEN. Reduction of PTEN dosage by EBV-miR-BART1 activated PTEN-dependent pathways, including PI3-K/Akt, FAK-p130 (Cas), and Shc-MAPK/ERK1/2 signaling, drove EMT, and consequently increased migration, invasion, and metastasis of NPC cells, highlighting the important role of PTEN in EBV-miR-BART-driven metastasis in NPC.^274^

#### EBV-miR-BART2-5p

Circulating EBV miRNA BART2-5p has been reported to act as a valuable biomarker for early detection and screening of NPC.^275^

#### EBV-miR-BART5-3p

The upregulation of EBV-miR-BART5-3p promoted the growth of NPC and gastric carcinoma cells. BART5-3p directly targeted the tumor suppressor gene, *TP53*, on its 3ʹ-untranslated region (3ʹ-UTR) and consequently downregulated CDKN1A, BAX, and FAS expression, leading to acceleration of the cell cycle progress and inhibition of apoptosis. BART5-3p also contributed to resistance to chemotherapeutic drugs and ionizing irradiation-induced p53 increases.^276^

#### EBV-miR-BART6-3p

EBV-encoded miR-BART6-3p inhibits EBV-associated cancer cell migration and invasion by targeting and down-regulating a novel lncRNA LOC553103, providing a potential novel diagnostic and treatment biomarker for NPC and other EBV-related cancers.^277^ Through proteomics analysis, stathmin (STMN1) was determined to be affected by EBV-miR-BART6-3p and LOC553103. LOC553103 directly binds to and stabilizes the 3ʹ-UTR region of *STMN1* mRNA. These results indicated that the EBV-miR-BART6-3p/LOC553103/STMN1 axis regulated the expression of cell cycle-associated proteins, which then inhibited NPC cell proliferation.^278^

#### EBV-miR-BART7-3p

EBV-miR-BART7-3p also targeted the human tumor suppressor PTEN, modulating PI3-K/Akt/GSK-3β signaling and eventually leading to high expression and nuclear accumulation of Snail and β-catenin, which favor EMT.^279^ Cai et al.^280^ identified *SMAD7* as a novel target gene of EBV-miR-BART7-3p in addition to the *PTEN* gene that was previously reported; and this viral miRNA suppressed SMAD7, leading to activation of TGF-β signaling and eventually enhanced the stemness of NPC cells. Also, the EBV-miR-BART7 increased responsiveness of NPC to radiation treatment by targeting GFPT1/TGF-β1 signaling. Thus, detection of EBV-miR-BART7 might be a useful indicator for monitoring NPC progression and predicting radio-therapeutic outcomes.^281^

#### EBV-miR-BART8-3p

EBV-miR-BART8-3p promotes NPC cell proliferation in response to irradiation in vitro and is associated with the induction of cell cycle arrest at the G2/M phase, which is a positive factor for the DNA repair after radiation treatment. Treatment with KU60019 or AZD6738 increases the radiosensitivity of NPC by suppressing the expression of p-ATM and p-ATR, indicating that EBV-miR-BART8-3p promoted radioresistance in NPC by modulating the activity of the ATM/ATR signaling pathway.^282^

#### EBV-miR-BART13-3p

Vesicle-bound EBV-miR-BART13-3p in circulation distinguished NPC from other head and neck cancers and asymptomatic EBV-infections. The high specificity of circulating EBV-miR-BART13-3p (97%) for NPC detection is in agreement with active secretion from NPC tumor cells. Thus, EV-bound EBV-miR-BART13-3p in circulation is a promising, NPC-selective, biomarker that should be considered as part of a screening strategy to identify NPC in endemic regions.^283^

#### EBV-miR-BHRF1-1

The EBV-encoded miR-BHRF1-1 is barely expressed in most NPC cells with EBV latent infection. miR-BHRF1-1 has been shown to be involved in TPA-induced accumulation of EBV lytic proteins and viral copies in late lytic cycle. The miR-BHRF1-1-potentiated induction of EBV lytic replication is accompanied by inhibition of p53 expression. These results suggested that the EBV original pathogen miR-BHRF1-1 is involved in the control of EBV late lytic replication by directly targeting the host *p53* gene.^284^

#### EBV-BART-lncRNAs

BART lncRNAs localize within the nucleus of EBV-infected cells and knockdown of BART lncRNAs substantially affected the expression of genes associated with cell adhesion, oxidoreductase activity, inflammation, and immunity. BART lncRNA is associated with the CBP/p300 complex and RNA polymerase II (Pol II) in the nucleus, suggesting that BART lncRNAs might mediate epigenetic regulation of gene expression by interacting with the chromatin remodeling machinery. EBV BART lncRNA expression modulates host gene expression and maintains EBV latency by interfering with histone methylation and acetylation processes. Thus, aberrant expression of affected host genes mediated by BART lncRNA might lead to immune evasion, progression, and metastasis of NPC.^285^ Verhoeven et al.^286^ showed that NF-κB activates the BART promoters and regulates expression of EBV-BART miRNAs and lncRNAs in NPC. The aberrant NF-κB signaling and expression of BARTs form an autoregulatory loop for maintaining EBV latency in NPC cells. Further exploration of how targeting NF-κB signaling interrupts EBV latency in NPC cells might reveal new options for NPC treatment.

#### EBV-encoded RNAs (EBERs)

Similar to EBV-BART miRNAs and lncRNAs, EBERs are another type of non-coding RNAs expressed by EBV and are highly expressed in all EBV infection programs. Owing to their most abundant viral transcripts and their own high abundance, in situ hybridization against EBERs still constitutes the gold standard for detecting EBV-infected cells. In contrast to the miRNAs, EBERs are confined to the nucleus and seem to interact with various RNA binding proteins. Transgenic overexpression of EBERs could lead to lymphoproliferations and, less frequently, B-cell lymphomas.^231^ Epstein–Barr-encoded RNA EBER2 increases CXCL8 expression, and this chemokine enhances spontaneous lytic replication levels in M81-infected B cells. The unique properties of M81 EBER2 could be ascribed to its unusually high expression level and to the ability of its single-stranded region to activate TLR7. Thus, M81 induces chronic inflammation in its target cells and resulting in increased virus production.^230^

#### EBV and exosomal miRNA

Interestingly, Zuo et al.^287^ revealed that aspirin was a promising drug for NPC therapy through its targeting of exosomal LMP1 (exo-LMP1) transfer and the regulatory effect of LMP1 on miR-203 expression. EBV could regulate its own tumorigenesis through the LMP1/NF-κB/exo-LMP1 axis, opening a new avenue for understanding the pathogenesis of this tumor virus and providing a rationale for the use of exo-LMP1 or exosomal miR-203 (exo-miR-203) in EBV-targeted therapy by aspirin in invasive NPC.

### The interactions between miRNA and lncRNA/circRNA

Long non-coding RNAs (lncRNAs) and circular RNAs (circRNAs) have been reported to act as important regulators and also to play crucial roles in NPC. LINC00460 was reported to be markedly increased in NPC tissues and cells compared to their corresponding controls. LINC00460 contributes to the progression of NPC by regulating the miR-149-5p/IL-6 signaling pathway, suggesting that LINC00460 could be regarded as a novel prognostic biomarker and therapeutic target in NPC diagnosis and treatment.^288^ CircRNA ZNF609 (circ-ANF609) and miR-188 have been, respectively, reported to play a pro-cancer and anticancer role in NPC. Circ-ZNF609 depletion represses proliferation and cell cycle transition, and induces apoptosis of NPC cells through modulation of the miR-188/ELF2 axis, providing potential targets for the therapy of NPC.^289^ Moreover, circCRIM1 is upregulated in highly metastatic NPC cells and tissues, and its overexpression promotes NPC cell metastasis and EMT. Mechanistically, circCRIM1 competitively binds to miR-422a and prevents the suppressive effects of miR-422a on its target gene *FOXQ1*, which finally leads to NPC metastasis, EMT, and docetaxel chemoresistance.^290^

### The combination of miRNA and transcriptome sequencing

Szeto et al.^291^ characterized mRNA and miRNA transcriptomes by RNA sequencing (RNA-Seq) of NPC model systems. They found 2812 genes and 149 miRNAs (human and EBV) to be differentially expressed in NP460, HK1, C666, and X666 with RNASeq and 533 miRNA-mRNA target pairs were inversely regulated in the three NPC cell lines compared to NP460 cells. Integrated mRNA/miRNA expression profiling and pathway analysis showed that extracellular matrix organization, beta-1 integrin cell surface interactions, and the PI3K/AKT, EGFR, ErbB, and Wnt pathways are potentially deregulated in NPC. This comprehensive characterization of mRNA and miRNA transcriptomes in NPC cell lines and xenografts provided insights on miRNA regulation of mRNA and valuable resources on transcript variation and regulation in NPC, which are potentially useful for mechanistic and preclinical studies.

### The link between miRNA/lncRNA and epigenetic regulation

Increasing evidence supports the role of members of the polycomb group (PcG) gene family in tumor development and progression. Using NPC as a disease model, a comprehensive analysis was undertaken to examine the clinical significance of EZH2 expression, identification of the cellular processes regulated by EZH2, and the mechanisms of its deregulated expression. The combination of global miRNA profiling in primary NPC specimens, and in silico analyses provided several candidate miRNAs that could regulate EZH2. miR-26a, miR-101, and miR-98, which were validated as bona fide regulators of EZH2 expression. In particular, miR-98 was under-expressed in samples from relapsed patient, strongly suggesting an important role for the miR-98 and EZH2 axis in NPC biology.^292^ miR-26a strongly reduced the expression of the *EZH2* oncogene in NPC cells. Similar to restoring miR-26a expression, EZH2 downregulation inhibited cell growth and cell-cycle progression, whereas EZH2 overexpression rescued the suppressive effect of miR-26a. These results indicated that miR-26a functions as a growth-suppressive miRNA in NPC, and that its suppressive effects are mediated chiefly by repressing EZH2 expression.^293^

### The clinical significance of ncRNA in EBV-associated tumors

#### miRNAs act as biomarkers

Circulating miRNAs have become reliable sources of noninvasive biomarkers for cancer diagnosis. miRNA expression analysis in circulating blood for the identification of novel signatures might assist in the early detection of NPC patients. Five miRNAs in serum, including let-7b-5p, miR-140-3p, miR-192-5p, miR-223-3p, and miR-24-3p were found to be significantly upregulated in NPC patients and are potential biomarkers for NPC detection.^294^ The dynamic changes in plasma miRNAs (miR-9-3p, miR-124-3p, miR-892b, and miR-3676-3p) after treatment reflect the outcome of the disease and have the potential to monitor recurrence and metastasis in patients with NPC.^295^ Additionally, the high expression levels of miR-548q and miR-483-5p in plasma, cell lines, and clinical tissues of NPC patients indicates their significant roles as potential biomarkers for NPC.^296^ Twenty-three plasma miRNAs with differential expression levels were selected for qPCR analysis on an independent set, including 100 NPC patients and 55 healthy controls. NPC patients with low concentrations of miR-483-5p and miR-103 had better prognosis for 5-year OS than those with high concentrations. In contrast, those with low concentrations of miR-29a and let-7c had poorer prognosis. Thus, differentially expressed plasma miRNAs as identified by next-generation sequencing could be helpful for predicting survival in NPC patients.^297^ Moreover, four-serum miRNAs (miR-22, miR-572, miR-638, and miR-1234) were found to be differentially altered and were used to construct a miRNA signature for NPC. This four-serum miRNA signature adds prognostic value to the TNM staging system and provides information for personalized therapy in NPC.^298^

Additionally, miR-451 is significantly downregulated in NPC cell lines and clinical tissues. Patients with low expression of miR-451 had poorer OS and disease-free survival than patients with high expression, and miR-451 is an independent prognostic factor in NPC as shown by multivariate Cox regression analysis.^299^ Three NPC subtypes (immunogenic, classical, and mesenchymal) have been identified as molecularly distinct and clinically relevant, of which the mesenchymal subtype (~ 36%) is associated with poor prognosis, characterized by suppressing tumor suppressor miRNAs and the activation of EMT. Out of the 25 most differentially expressed miRNAs in the mesenchymal subtype, miR-142, miR-26a, miR-141, and let-7i had significant prognostic power for prediction of distant metastasis.^300^

EBV-associated gastric carcinoma (EBVaGC) is the most common malignancy caused by EBV infection. EBVaGC has definite histological characteristics similar to gastric carcinoma with lymphoid stroma. Clinically, EBVaGC has a significantly low frequency of lymph node metastasis compared with EBV-negative gastric cancer, resulting in a better prognosis. EBVaGC harbors a DNA methylation phenotype, PD-L1 and PD-L2 overexpression, and frequent alterations in the *PIK3CA* gene.^301^ In a study aiming to determine the potential utility of circulating cell-free EBV DNA as a biomarker for the detection and/or monitoring of therapeutic response in patients with EBVaGC, Shoda et al.^302^ showed that plasma EBV ratios were significantly correlated with the size of EBVaGC tumors. Patients with EBVaGC might have a better prognosis, but circulating cell-free EBV DNA had no or little impact on prognosis. In addition, repeated assessment of the plasma EBV ratio in EBVaGC showed a decrease in plasma EBV DNA after treatment and increase during tumor progression/recurrence.

#### ncRNAs and therapy-resistance

For the roles of ncRNA in chemotherapy resistance, miR-374a was observed to reduce NPC cell proliferation, migration, invasion, metastasis, and chemotherapy drug cisplatin (DDP) resistance through the miR-374a-CCND1-pPI3-K/AKT-c-Jun feedback loop.^303^ MiR-296-3p is negatively regulated by nicotine and directly targets MK2-induced Ras/Braf/ERK/Mek/c-Myc or PI3K/AKT/c-Myc signaling to suppress NPC cell proliferation, metastasis and chemotherapy resistance to DDP treatment.^304^ Also, a tumor suppressor miRNA, miR-3188, could directly target the mTOR-p-PI3K/AKT-c-Jun pathway and participate in FOXO1-mediated repression of cell growth, tumorigenesis, and NPC chemotherapy resistance to 5-FU.^305^

For the roles of ncRNA in radiotherapy resistance, a sequence polymorphism rs4919510C > G in miR-608 was reported to act as a simple marker to predict locoregional recurrence (LRR) in patients with radiotherapy-treated NPC.^306^ In addition, miR-19b-3p contributes to the radio-resistance of NPC by activating the TNFAIP3/NF-κB axis, and miR-19b-3p is a determinant of NPC radio-response and might serve as a potential therapeutic target in NPC treatment.^307^ MiR-138-5p enhances radio-sensitivity of NPC cells by targeting EIF4EBP1.^308^ MiR-483-5p decreases the radio-sensitivity and radiation-induced apoptosis of NPC cells by targeting DAPK1.^309^ MiR-504 affects the radio-resistance of NPC by down-regulating the expression of NRF1 and disturbing mitochondrial respiratory function through TFAM and OXPHOS complexes I, III, and IV. The serum miR-504 expression levels of NPC patients are elevated during different weeks of radiotherapy and correlate with tumor, lymph nodes, and metastasis (TNM) stages and total tumor volume. This indicates that high serum miR-504 expression levels are associated with tumor radio-resistance and poor radio-therapeutic effects. Therefore, miR-504 might become a promising biomarker of NPC radio-resistance and targeting miR-504 might improve tumor radiation response.^310^ Additionally, LncRNA ANCR promotes NPC cell growth and radiation resistance by repressing the expression of PTEN. This regulation relies on ANCR-mediated EZH2 binding and epigenetic regulation on the *PTEN* promoter.^311^ CircRNA_000543 knockdown sensitized NPC cells to irradiation by targeting the miR-9/platelet-derived growth factor receptor B (PDGFRB) axis. Thus, circRNA_000543 might be a potential therapeutic target for radio-resistant NPC.^312^

## Radiochemotherapy in EBV-associated tumors

Radiotherapy (RT) alone is a good option for NPC patients with stage I disease, which is based on the eighth edition of the American Joint Committee on Cancer (AJCC) staging system.^313^ According to the National Comprehensive Cancer Network guidelines, the efficacious choice for patients with locoregionally advanced NPC (Stage II–IVa) is RT combined with chemotherapy. Depending on the tolerance and heterogeneity of patients, three regimens are used to treat locoregionally advanced NPC, including concurrent chemotherapy, adjuvant chemotherapy, and induction chemotherapy. Adjuvant chemotherapy and induction chemotherapy play a pivotal role in the development of concurrent chemotherapy, bringing more benefits to patients’ OS and PFS.^314^

### Molecular-targeted therapy combined with chemotherapy

Cisplatin is the paramount and classical first-line concurrent chemotherapy drug, compared with fluorouracil, oxaliplatin, or nedaplatin. Clinical treatment helped popularize cisplatin as a backbone drug for most solid tumor therapeutic schedules. It has been first approved by Food and Drug Administration (FDA) to treat testicular cancer and bladder cancer in 1978. However, chemotherapy drugs are limited by their harsh toxicity and development of drug resistance in the process of treatment.^315^ Cisplatin-based chemotherapy benefits only 10%-20% of patients with squamous cell carcinomas of the head and neck (HNSCC). NPC is a subtype of HNSCC that shares similar characteristics.^11,316^ Currently, with the rapid progress of molecular diagnostic therapies, the development of more targeted drugs for the treatment of NPC to improve the prognosis of patients has become a promising prospect.

Several clinical trials have shown good contributions to patients’ OS and PFS by molecular-targeted drugs combined with concomitant radiochemotherapy in locoregionally advanced NPC. Epidermal growth factor receptor (EGFR) was defined as a driving oncogene with high mutations rate in many tumors, but not in NPC. EGFR is overexpressed in almost 80% of NPCs and is enhanced by the EBV-encoded oncoprotein, LMP1. Our previous studies have shown that nuclear EGFR/signal transducer and activator of transcription 3 (STAT3) can directly bind to and activate the *cyclin D1* promoter under the regulation of LMP1, favoring cell proliferation and cycle progression.^317,318^ Monoclonal antibodies against EGFR, such as cetuximab and nimotuzumab are dominant among molecular-targeted therapies. Three phase II clinical trials (NCT01271439, NCT00700440, NCT01712919) have aimed to evaluate the toxicity and efficacy of cetuximab combined with chemotherapy in locoregionally advanced NPC. A phase II study showed the value of an intravenous immunoglobulin G1 monoclonal antibody (cetuximab) in combination with carboplatin in patients with recurrent or metastatic NPC.^319^ Significantly, another phase II study of an orally active EGFR tyrosine kinase inhibitor (gefitinib) in combination with cisplatin offers promising efficacy as shown by evaluation of plasma Epstein–Barr virus DNA and a manageable safety profile for patients with metastatic or locoregionally recurrent NPC.^320^ All of these trials will contribute to obtain a thoroughly understanding of the role of EGFR combined with concurrent chemotherapy in NPC. Moreover, vascular endothelial growth factor (VEGF) is another molecular target that is more active in combination chemotherapy treatment. A completed Phase II clinical trial (NCT00408694) covered the use of a monoclonal antibody (bevacizumab) together with cisplatin and fluorouracil aimed at stopping the growth of NPC by blocking blood flow to the tumor by targeting VEGF.

### Biomarkers of chemotherapy for EBV-associated tumors

Additionally, numerous other molecular-targeting methods are in clinical trials, including interruption of compound and gene modifications. A novel and potent imidazo[4,5-c] quinolone derivative (NVP-BEZ235), which has entered phase I/II clinical trials in patients with advanced solid tumors, has a potential role in cisplatin-sensitive cancers by dual targeting of the PI3-K (phosphatidylinositol 3-kinase) and mTOR (mammalian target of rapamycin) kinases for NPC treatment.^321^ The potential ability of NPC stem cells to evade the apoptotic pathways can make them resistant to chemotherapy due to their potential ability to evade cell death. One study showed that overexpression of the inhibitory apoptotic protein protein (IAP) family in NPC stem cells may play a role in maintaining NPC stem cell characteristics. Targeting cancer stem cells (CSCs) by a novel antagonist of IAPs, APG-1387, induced autophagic degradation of sex-determining region Y (SRY)-box 2 (Sox2), suggesting that APG-1387 combined with chemotherapy might be a novel therapeutic strategy for the treatment of NPC.^322^ The search for anticancer agents originating from natural compounds has been a booming area of research in recent years due to their perceived low toxicity. Asiatic acid (AA), extracted from *Centella asiatica*, was found to have anticancer activity against various cancers, and appears to overcome cisplatin resistance by triggering apoptosis through the induction of p38 phosphorylation.^323^

Interfering with drug-resistant molecules through genetic modification is a common therapeutic strategy. Tumor cells can evade the killing effects of chemotherapy through molecular mechanisms of cell cycle blockade and DNA damage repair. The new 6q27 tumor suppressor disheveled-associated binding antagonist of beta-catenin 2 (DACT2), which is frequently silenced by CpG methylation, sensitizes NPC cells to paclitaxel and 5-FU toxicity through β-catenin/ Cdc25c signaling and G2/M arrest.^324^ Aryl hydrocarbon receptor nuclear translocator-like (ARNTL) hypermethylation promotes tumorigenesis and inhibits cisplatin sensitivity by activating cyclin-dependent kinase 5 (CDK5) transcription in NPC.^325^ Nuclear factor with BRCT domain protein 1 (NFBD1) functions in cell cycle checkpoint activation and DNA repair following DNA damage, which accompanying by cisplatin or 5-fluorouraci resistance in NPC.^326^ Indeed, the epithelial–mesenchymal transition (EMT) is considered to be the main driver of chemotherapy resistance in NPC. For example, forkhead box C2 (FOXC2)-mediated EMT may be an important mechanism through which cancer cells acquire and maintain drug resistance.^327^ Another report revealed that matrix metalloproteinase 1 promotes tumorigenesis and inhibits NPC’s sensitivity to 5-fluorouracil.^328^ Our previous study showed that EBV-LMP1 activates PKC to promote the nuclear entry of annexin A2 and induce DNA synthesis and cell proliferation.^329^ Moreover, human NPC cells with high ANXA2 expression can potentially resist cisplatin or 5-fluorouracil, docetaxel, and vincristine by upregulating EMT-associated signaling proteins. Therefore, annexin A2 might be a biomarker to evaluate the resistance of NPC during chemotherapy treatment.^330^ Recently, a growing number of studies show that triggering an immune response in cancer patients can make chemotherapy work better. Bone marrow stromal cell antigen 2 (BST2) is well-known as a natural immune factor in viral infections and is highly expressed in a majority of tumors. Knockdown of BST2 with siRNA leads to cisplatin resistance by activating the NF-κB signaling pathway and enhancing the expression of anti-apoptotic genes, such as Bcl-XL and livin.^331^ The CD271-associated protein, brain-expressed X-linked 3 (BEX3), is accompanied with high octamer-binding transcription factor 4 (OCT4) expression in cisplatin-resistant NPC, suggesting that targeting BEX3 in combination with cisplatin could have a synergistic effect on NPC.^332^ In addition, some EBV-driven treatments are forecasted to be effective only when lytic viral replication occurs. Cyclophosphamide can induce the lytic phase of EBV infection and is quite effective in reducing the number of EBER-expressing tumor cells within 5 days.^333^ DNA viral reactivation due to histone deacetylase inhibitor-induced immunosuppression or chromatin remodeling. In a phase II clinical trial of the HDAC inhibitor, romidepsin, in patients with T-cell lymphoma, 2 of 120 treated patients developed secondary EBV-associated lymphoproliferations. This trial suggests that reactivation of latent EBV is well characterized as an outcome of the immune suppression associated with systemic chemotherapy.^334^

All of these studies were aimed at confirming molecules related to chemoresistance. Notably, elucidating the molecular mechanisms of chemoresistance would benefit in discovering a personalized and accurate therapeutic approach for EBV-associated tumors.

### EBV DNA as a biomarker in chemotherapy

Emerging evidence supports a role for EBV in NPC treatment. Significantly, an unfavorable EBV DNA response after induction chemotherapy or at the midpoint of RT is also shown to be an adverse prognosticator for clinical outcome. The plasma EBV DNA load at induction chemotherapy (ICT) completion (postICT-DNA) is a powerful and early outcome predictor in locoregionally advanced NPC, allowing comparison of the prognostic value of postICT-DNA and post-chemoradiation therapy (CCRT) DNA (postRT-DNA), which could facilitate further risk stratification and early treatment modification.^335^ Detectable EBV DNA after NACT in advanced-stage NPC is correlated with poor clinical outcome.^336^ EBV DNA level and tumor response after NACT may complement each other for assessing outcome in NPC patients who have received both NACT and CCRT. Two clinical trials (NCT02135042, NCT03668730) showed that EBV DNA could be used to identify patients at high risk for treatment failure and to perform further risk stratification, early treatment modification, or both, before the implementation of CCRT.^243,337^ Plasma EBV DNA is also of predictive value for prognosis in metastatic/recurrent NPC patients undergoing palliative chemotherapy.^338^ Plasma clearance of cell-free Epstein–Barr virus (pEBV) DNA could predict survival and subsequent response in patients with advanced or recurrent NPC. This dual endpoint is an innovative tool for assessing early drug responses.^339^ Furthermore, the influence of cumulative doses of cisplatin on the clinical outcome in NPC patients who received intensity-modulated radiotherapy (IMRT) has been investigated. A study suggested that the optimal cumulative dose of cisplatin in low-risk group patients (EBV DNA <4000 copies/ml) was adjusted by assessing OS.^340^ After initial treatment of advanced stage (stage III–IV) extranodal NK/T-cell lymphoma with cisplatin, dexamethasone, gemcitabine, or pegaspargase (DDGP), OS was found to be superior in the EBV-DNA-negative group compared to the EBV-DNA-positive group.^341^

### EBV-activated chemotherapy-resistant signaling

The DNA damage response (DDR) is a surveillance mechanism to monitor genome integrity and coordinate different aspects of cellular response, including cell cycle progression, gene transcription, and DNA repair. DDR is dysregulated by EBV infection with EBV-encoded viral proteins.^99^ Studies have shown that EBV infection activates DNA damage checkpoints by promoting the phosphorylation of the ATM and CHK2 pathway. Phosphorylation of the ATR/checkpoint kinase 1 (CHK1) pathway proteins and gradually activated along with the duration of the EBV exposure in NPC cell lines. EBV activation of the ATR-mediated DNA damage response results in chemotherapy resistance to CDDP and 5-FU in NPC. Accordingly, ATR knockdown may serve as an effective treatment strategy for chemotherapy-resistant, EBV-positive NPC.^342^ Upregulation of NOTCH ligands (JAG1 or DLL4) and effector (HEY1) in the majority of EBV-positive tumor lines and primary tumors has been observed. NOTCH3 knockdown highly enhances the sensitivity of NPC cells to cisplatin treatment. Targeting NOTCH3 signaling may serve as a potential therapeutic approach for treating patients suffering from EBV-associated NPC.^343^

Recently, we revealed that the IC_50_ of cisplatin in EBV-LMP1-positive cells was significantly higher than that in EBV-LMP1-negative cells. The mechanisms behind this chemoresistant phenotype are closely related to the numerous EBV-mediated oncogenic signaling pathways, providing new approaches for preventing and reversing drug resistance in NPC. For example, EBV-LMP1 remodels the mitochondria by regulating the AMPK/Drp1 and cyclin B1/Cdk1/Drp1 axes,^101^ or decreases TRAIL-induced apoptosis by activating the PI3-K/AKT/FOXO3a signaling pathway.^344^ In addition, EBV-LMP1 modulates MAPK-mediated Op18/stathmin signaling to induce cell cycle progression and promote tumorigenesis.^345^ SiRNA-mediated silencing of stathmin may enhance the efficacy of paclitaxel in the treatment of NPC.^346^

EBV-miR-BART22 not only promoted tumor stemness and metastasis, but also enhanced the resistance to cisplatin (DDP) in vitro and in vivo. Cinobufotalin markedly reversed EBV-miR-BART22-induced cisplatin resistance by stimulating MAP2K4 to antagonize the non-muscle myosin heavy chain IIA/ glycogen synthase 3β/β-catenin signaling pathway.^347^

In summary, molecular-targeted therapy improves chemosensitivity and has the potential to interfere with tumor metastasis. EBV-mediated signaling pathways are important mechanisms of the phenotypic behavior of treatment resistance in EBV-related tumors. EBV DNA has been demonstrated to play a role as a prognosis biomarker to evaluate the efficacy of treatment in chemotherapy-based patients.

### Radiation therapy in EBV-associated tumors

So far, the widespread application of intensity-modulated radiotherapy and optimization of chemotherapy strategies (induction, concurrent and adjuvant) are the main strategy in NPC clinical therapy and have contributed to improved survival with reduced toxicities.^11^ However, radiation resistance has been a major hurdle that limits therapeutic efficacy and results in recurrence.^11,208^

#### ROS-mediated oxidative stress in radiation therapy

So far, several specific signaling pathways contribute to cells gaining resistance against ionizing radiation. Among these signaling pathways, ROS play a significant but paradoxical role acting as a “double-edged sword” to regulate cellular response to radiation. On the one hand, ROS is a critical mediator of IR-induced cell killing. When ROS levels rise beyond a tolerable limit inside tumor cells, apoptosis is initiated and ROS-induced cell death may be either direct or indirect.^348^ High levels of ROS cause a cluster of lesions to form within DNA that are difficult to repair and lead directly to cell death. Indirect cell death involves altered cellular homeostasis and modified signaling pathways ultimately leading to apoptosis.^349^ On the other hand, ROS is a critical regulator of IR-resistant pathways.^223^ The tumor cell stemness, antioxidant enzymes expression and inflammation are the reason that related to the ROS-mediated IR-resistant.^236^ Presently, some biomarkers have been identified that enhance the radiosensitivity of NPC, and include COX-2, EGFR, Bcl-2, and VEGF. These markers were associated with cell cycle, apoptosis, angiogenesis, and DNA repair.^350^

#### LMP1 contributes to radioresistance

EBV-latent membrane protein 1 (LMP1), a driving oncogene in NPC, expresses in almost all primary NPC tissues.^227^ Our group have revealed that EBV (LMP1)-positive cells show a higher survival fraction were resistant to radiation therapy. The mechanism was that LMP1 appears to activate several oncogenic signaling axes, including LMP1/JNKs/c-Jun/HIF-1/VEGF,^351^ LMP1/NF-κB/ATM,^352^ LMP1/Akt/hTERTs,^353^ LMP1/PI3K/Akt/GSK3β/c-Myc/HK2,^98^ and LMP1/DNA-PK/AMPK/DDR,^99^ which mediate therapeutic resistance. Recently, we found that reactivation of EBV by LMP1-induced high oxidative stress could lead to radioresistance of NPC cells both in vitro and in vivo, suggesting that EBV reactivation plays an important role in NPC resistance (unpublished data). As LMP1 is an attractive and promising target for EBV-positive NPC, our group successfully developed a DNAzyme (DZ1) that was engineered to specifically target the LMP1 mRNA.^255^ DZ1 treatment can reverse malignant phenotypes caused by LMP1 and increase the radiosensitivity of NPC, indicating the potential for DZ1 therapeutic approaches for the treatment of EBV-related cancers.^99,255,354,355^

#### Other targets contribute to radioresistance

Furthermore, there are several other signaling pathways that are related to radioresistance. For example, the forkhead box O (FOXO) family of proteins is an important transcriptional regulator of pivotal proteins associated with many diverse cellular functions.^356^ Among them, FOXO3a has been extensively studied because of its unique role in the regulation of cell proliferation, apoptosis, metabolism, stress management, and longevity.^357^ In NPC, silencing FOXO3a promotes tumor radioresistance of NPC in vitro and in vivo through inducing EMT and activating Wnt/β-catenin signal pathway. It is supposed that FOXO3a can be a novel and reliable NPC marker and a potential therapeutic target against NPC.^358^ Guo et al.^359^ reported genetic variations in the PI3K/PTEN/AKT/mTOR pathway are associated with distant metastasis in nasopharyngeal carcinoma patients treated with intensity-modulated radiation therapy. Zhang et al.^360^ reported PTEN can regulate the radiation-resistant cancer stem-like cell properties through activity of nuclear β-catenin in nasopharyngeal carcinoma. In addition, inhibition of the Numb/Notch signaling pathway increases radiation sensitivity in human nasopharyngeal carcinoma cells.^361^ In this part, we primarily discuss the regulation of oxidative stress by EBV, the role of EBV lytic reactivation in EBV-associated diseases and the following radiation therapy. A better understanding of the relationship between them may lead to develop novel prevention and therapeutic strategies for EBV-associated diseases and facilitate the development of novel strategies against radiation-resistant tumors.

## Immunotherapy for EBV-associated diseases

The expression of the EBV antigen and the dense interstitial infiltration of immune cells provide a unique and attractive target for immunotherapy in EBV-related tumors.^7,11,362–364^ The immunotherapy of EBV-associated malignancies primarily includes EBV-targeted vaccination and adoptive T-cell therapy, as well as immune checkpoint blockade, some of which show good antitumor activity in early clinical studies.

### EBV-related vaccines

Two main types of EBV-related vaccines exist, including one that is preventive and the other that is therapeutic targeting EBV-related life cycle antigens. Gp350 belongs to the product of the virus lytic stage and is relatively easier to produce neutralizing antibodies, which makes it a candidate antigen for prophylactic vaccines.^365^ Most studies show that the gp350 prophylactic vaccine does not achieve satisfactory clinical results, which may be closely related to the number of clinical samples, research time, appropriate adjuvants, and vaccination time.^366,367^ No experimental data are yet available supporting the idea that the gp350 vaccine can prevent the occurrence of NPC, but some studies have confirmed that high levels of neutralizing antibodies are associated with a lower risk of NPC.^368^

The EBV latent proteins, EBNA1, LMP1, and LMP2, are continuously expressed in EBV-related tumor cells. These proteins are key factors in promoting the transformation of normal cells into tumor cells. Thus, therapeutic vaccines against these proteins has been widely studied in clinical trials and some results have been achieved^369^ (Table 8). EBNA1 and LMP2 generate the most robust T-cell immunity. Interestingly, the oncogenic protein, LMP1, is a very weak immunogen, generating very low levels of CD8^+^ T-cell immunity as both a standalone vaccine and as a part of a trivalent vaccine cocktail.^370^ LMP2 has a large number of CD8^+^ T-cell epitopes and a limited number of CD4^+^ T-cell epitopes, and vaccines against LMP2 have also been actively developed. The current clinical research on therapeutic vaccines for EBV- associated tumors focuses on NPC. Results from an experiment of transferring LMP2 antigen (rAd5-EBV-LMP2) by using recombinant adenovirus vector, phase I clinical trials of 3 different doses in patients with advanced NPC (D0, D7, D14, and D28), showed that the proportion of LMP2-specific CD3^+^ and CD4^+^ cells in peripheral blood of immunized patients increased in a dose-dependent manner within 28 days.^371^ In a phase II clinical trial evaluating the ability of dendritic cell (DC) vaccines to target LMP1 and LMP2 antigens in patients with advanced metastatic NPC, patients were vaccinated using adenovirus-transduced autologous DCs encoding truncated LMP1 (ΔLMP1) and full-length LMP2 (Ad-ΔLMP1-LMP2).^372^ Three patients had clinical responses showing specific T-cell activation, one partial response (7.5 months) and two stable disease (6.5 and 7.5 months), which means that Ad-ΔLMP1-LMP2 transduction of DC can be successfully generated and safely administered to patients with advanced NPC.^372^ Owing to limited efficacy, future studies should focus on administering DC vaccines with greater efficacy to subjects with less tumor burden. The carboxyl terminal and full-length LMP2 and EBNA1 were transmitted by a MVA vector as a vaccine to obtain fusion proteins (MVA-EL). The vaccine effectively expanded EBNA1 and LMP2-specific CD4^+^ and CD8^+^ cells from peripheral blood lymphocytes of healthy serum EBV-positive individuals in vitro*.*^373^ A phase I clinical trial of MVA-EL vaccine was conducted in Hong Kong, comprising NPC patients who were in remission at least 12 weeks after initial treatment and received three intradermal MVA-EL immunizations. The results showed that the vaccine was well tolerated and had no dose-dependent toxicity, and the T-cell response to one or two vaccine antigens tripled in 15 of the 18 patients who received treatment.^374^ Phase I clinical trials of the MVA-EL vaccine comprising 16 NPC patients in the UK also showed that 8 of 14 patients who received treatment had increased CD4^+^ and CD8^+^ T-cell responses to one or both antigens, and immunophenotypic analysis confirmed that vaccination induced differentiation and functional diversity of EBNA1- and LMP2-specific CD4^+^ and CD8^+^.^375^ Owing to the satisfactory results of two phase I trials, the Cancer Research UK will further evaluate adult patients with NPC (NCT01800071).

**Table 8:** Clinical trials of EBV-related therapeutic vaccines

Ref.	Conditions	Interventions	Enrollment	Clinical outcome
NCT01800071	EBV-positive NPC	MVA-EBNA1/LMP2 vaccine	22	Immune memory and recall response to MVA-EBNA1/LMP2 vaccination, Measurement of EBV genome levels
NCT01094405	EBV-positive NPC	Recombinant Epstein–Barr virus (EBV) vaccine	25	Objective Response Rate (ORR), Duration of Response (DR), Progression-free survival (PFS), Overall survival (OS)
NCT00078494	Recurrence NPC	EBV-LMP-2 peptide	99	Evaluate how the vaccines affect immune system cells
NCT00478062	After Initial Therapy of Hodgkin’s Lymphoma	Hodgkin’s antigens-GM-CSF-expressing cell vaccine	35	Determine immunologic responses
NCT01147991	EBV-positive cancers	EBNA1 C-terminal/LMP2 chimeric protein	16	To determine safety and immunologic responses
Hui et al.^374^ NCT01256853	EBV-positive NPC	MVA-EL, which encodes an EBNA1/LMP2 fusion protein	18	Toxicity of MVA-EBNA1/LMP2 vaccine, T-cell responses, and assess the changes of EBV genome
Si et al.^371^	EBV-positive NPC	Recombinant adenoviral vaccine expressing EBV-LMP2 protein (rAd5-EBV-LMP2)	24	Proportion of CD3^+^ CD4^+^ cells in peripheral blood
Chia et al.^372^	EBV-positive NPC	DCs transduced with adenovirus encoding a truncated LMP1 (DeltaLMP1) and full-length LMP2 (Ad-DeltaLMP1-LMP2)	16	Detected in tLMP1/2-specific T cell
Lin et al.^372^	EBV-positive NPC	Autologous DCs pulsed with HLA-restricted epitope peptides from LMP2	16	CD8^+^ T-cell responses
Li et al.^466^	EBV-positive NPC	Autologous DCs pulsed with HLA-restricted epitope peptides from LMP2	16	Patients responded to LMP2A peptides Serum EBV-DNA level

The main goal of vaccine development is to prevent primary infection, the establishment of a latent period, and subsequently the development of EBV-associated tumors. To date, developing a vaccine against this virus has been difficult. Most vaccines are primarily designed to induce a protective antibody response, which is considered too risky even when used in an attenuated form due to the inherent carcinogenicity of EBV, which is a critical problem in developing a vaccine against the virus.^376^ Therefore, new therapeutic vaccines should be either recombinant viral vectors capable of inducing EBV-specific cellular immune responses or novel recombinant viral glycoprotein preparations capable of eliciting more efficient EBV-specific antibody responses.^377–379^

### Adoptive immunotherapy in EBV-related cancers

EBV expresses potential tumor-associated virus antigens, making it a candidate focus for successive cell-based immunotherapy (CBI). Various preclinical and clinical studies have explored the application of cytotoxic T cells (CTLs), tumor-infiltrating lymphocytes (TIL), natural killer (NK) cells, and dendritic cells (DC) in the treatment of refractory and locally advanced NPC. Adoptive immunotherapy of EBV-associated cancers mainly works by bypassing antigen presentation steps, including direct activation of effector cells, especially CTLs and NK cells (Table 9).

**Table 9:** Cellular-based immunotherapy clinical trials in EBV-related cancers

NCT number	Study	Interventions	Phases	Enrollment
*CTL trails*				
NCT00516087	LMP1- and LMP2-specific CTLs to patients with EBV-positive NPC (NATELLA)	Genetically modified CTLs	I	23
Louis et al.^381^ NCT00078546	EBV-specific CTLs following CD45 antibody to patients with Epstein–Barr virus (EBV) + NPC	EBV-specific CTL Infusion and Anti CD45 monoclonal antibody	I	12
NCT00431210	Epstein–Barr virus-specific immunotherapy for NPC	EBV-specific adoptive T-cells	I	28
NCT02578641	Evaluating chemotherapy and immunotherapy for advanced NPC	Autologous EBV-specific cytotoxic T cells	III	330
NCT00834093	Study of Epstein–Barr virus-specific immunotherapy for NPC	EBV-Specific Immunotherapy	II	20
NCT02065362	TGF-β-resistant cytotoxic T-lymphocytes in treatment of EBV-positive NPC/RESIST-NPC	NPC-specific T cells	I	14
NCT01195480	CD19-CAR immunotherapy for childhood acute lymphoblastic leukemia	EBV-specific CTLs	I/II	29
NCT00058604	Prevention and treatment of Epstein–Barr virus (EBV) lymphoma following a solid organ transplant using EBV-specific cytotoxic T lymphocytes (CTLs)	EBV-specific CTLs	I	12
NCT00058617	Epstein–Barr virus (EBV)-specific cytotoxic T-Cells, relapsed lymphoma, ANGEL	EBV-specific CTLs	I	13
NCT01447056	Most closely HLA-matched CTLs for relapsed Epstein– Barr virus (EBV)-associated diseases (MALTED)	LMP-specific T cells	I	37
NCT01498484	Therapeutic effects of Epstein–Barr virus immune T-lymphocytes derived from a normal HLA-compatible or partially matched third-party donor in the treatment of EBV lymphoproliferative disorders and EBV-associated malignancies	EBV-specific T cells	II	87
NCT01555892	Cytotoxic T-lymphocytes for EBV-positive lymphoma, GRALE	EBV-specific T cells	I	136
NCT03988582	Safety and effectiveness of EBV-specific cytotoxic T cells for the treatment for EBV lymphomas or other EBV-associated malignancies	EBV-specific T cells	II	79
Bollard et al.^385^ NCT00062868	LMP-specific T-cells for patients with relapsed EBV-positive lymphoma (ALCI)	LMP1/2 CTLs	I	74
*NK-cell trails*				
NCT00717184	A Pilot study of autologous ex vivo activated NK-cell infusion in the treatment of metastatic NPC	Interleukin, NK cells	I	Unknown
NCT02507154	Reactivating NK cells in treating refractory head and neck cancer	Cetuximab + NK cells	I/II	31

#### Adoptive T-cell therapy

Adoptive T-cell therapy against EBV antigens includes stimulating autologous CD8^+^ T cells in vitro and then infusing them intravenously into the patients. This treatment strategy has several advantages. First, T cells have a high affinity for binding to EBV antigens and can be expanded in vitro, which can easily reach the number needed for clinical treatment.^380^ In addition, patients can receive other treatments before transfusion of T cells to remove tumor-related suppressor immune cells, which is conducive to the growth and expansion of injected lymphocytes in vivo. Some clinical experiments have shown that adoptive T-cell therapy targeting EBV antigens has an exciting effect. Eight patients with refractory NPC were injected with increasing doses of a CD45 mAb, before receiving EBV-CTL. Results showed that after injection of the CD45 mAb, EBV peptide-specific T cells increased 2.8-fold within 8 weeks. In this study, the clinical benefit rate was 37.5%.^381^ Second, Secondino et al.^382^ conducted a similar study on 11 patients with refractory NPC treated with cyclophosphamide and fludarabine. Based on the standard of tumor regression or stability of more than 4 months, the disease control rate was 54.5%. Chia et al.^383^ further evaluated the effect of EBV-CTL infusion into patients with locally advanced NPC and treated with the standard first-line chemotherapy of gemcitabine and^384^ carboplatin. Most of the patients showed positive reactions, such as decreased tumor growth rate, reduced tumor size, and an extended period of disease stabilization. This study showed that in the treatment of patients with metastatic and/or locally recurrent NPC, the median OS time was 29.9 months, and the 2-year OS rate was 62.9%.^383^ Importantly, the observed clinical benefits were directly related to the infusion of LMP2-specific T cells in CTL, as evidenced by a significant improvement in OS compared with patients who received CTL that lacked LMP2 specificity. In response to these encouraging results, the third phase of the EBV-CTL study is currently under way (NCT02578641). Similarly, Bollard et al.^385^ studied 28 patients with high-risk or multiple recurrent EBV-positive lymphomas treated with LMP-CTLs adjuvant with a median remission period of 3.1 years after CTL infusion. Of the 21 patients with recurrent or refractory lymphomas, 13 had clinical responses and 11 had complete responses. LMP-specific T cells and non-viral tumor-associated antigens were detected in peripheral blood within 2 months of CTL infusion.

#### NK cells therapy

NK cells have the inherent ability to distinguish between viral infection and malignant transformed cells without prior antigen sensitization. Some ongoing clinical trials have also attempted to use NK cells as an alternative to adoptive cell therapy for NPC.^386^ Compared with CTL, NK cells have a much shorter lifespan and do not need suicide vectors to overcome the overexpansion of metastatic cells.^387^ However, NK cells account for only a small portion of the human lymphocyte population (5–20%), and their distorted phenotypic and functional impairment during cancer progression challenges the activation and expansion of clinical-grade purified NK cells in vitro to reach the optimal number of clinical transfusions.^388^ Therefore, several methods have been developed and optimized, including the addition of cytokines for treatment and long-term cultures,^389^ and the use of peripheral blood mononuclear cells, K562 cells, and lymphoblastoid cell lines (LCL) transformed by EBV as feeder cells.^390^ In addition, tumor-directed NK-cell immunotherapy can be accomplished by genetic engineering of NK cells expressing tumor antigen receptors, or by combining with monoclonal antibodies that give priority to killing tumors through extracellular Fc receptor-mediated antibody-dependent cytotoxic (ADCC).^391^ At present, phase I/II clinical trials of highly active NK cells and expanded NK cells combined with the EGFR monoclonal antibody, cetuximab, in the treatment of refractory cancers are under way (NCT02507154).

### Immune checkpoint inhibitor therapy in EBV-associated cancers

Overexpression of programmed death ligand 1/2 (PD-L1/2) is a common event in EBV-associated tumors, suggesting the importance of immune evasion in tumorigenesis, which establishes the theoretical basis for the use of immune checkpoint inhibitors in EBV-positive tumors.^29^ Preliminary clinical data suggest that PD-1 inhibitors such as nivolumab, pembrolizumab, and avelumab are a promising therapeutic strategy for the treatment of certain EBV-associated tumors, particularly in recurrent or metastatic (R/M) EBV-positive tumors in combination with other treatment methods (e.g., radiotherapy, chemotherapy, or molecular-targeted drugs) showing promising clinical results^392,393^ (Table 10).

**Table 10:** Clinical trials on the use of PD-1 inhibitors for recurrent/metastatic EBV-related tumors

Trial	Phase	Setting	Enrollment	Agent	Combination therapy	Efficacy
Ma^399^ NCT02339558	II	R/M NPC	40	Nivolumab	NO	Completed
NCT02054806	Ib	R/M NPC	44	Pembrolizumab	NO	Completed
NCT02875613	II	R/M NPC	39	Avelumab	NO	Ongoing
NCT03707509	III	R/M NPC	250	Camrelizumab	Gemcitabine, Cisplatin	Ongoing
NCT02605967	II	R/M NPC	114	PDR001	Gemcitabine,capecitabine ordocetaxel	Ongoing
NCT03813394	I/II	R/M NPC	48	pembrolizumab	bevacizumab	Ongoing
NCT02611960	III	R/M NPC	124	Pembrolizumab	Gemcitabine, capecitabine	Ongoing
NCT03854838	II	R NPC	25	Tolipalimab	IMRT	Ongoing
NCT03907826	III	R NPC	212	Toripalimab	IMRT	Ongoing
NCT03930498	II	R NPC	43	Toripalimab	Cisplatin and Gemcitabine IMRT	Ongoing
NCT03755440	II	M GC	20	SHR-1210	NO	Ongoing
NCT03015896	I/II	R NHL/HL	102	Nivolumab	Lenalidomide	Ongoing
NCT02973113	I	R/R HL	8	Nivolumab	EBVST Cells	Ongoing
NCT02950220	I	R/R NHL	–	Pembrolizumab	Ibrutinib	Completed
Fang^403^ NCT03121716	I	R/M NPC	20	SHR-1210	Gemcitabine Cis-platinum	Completed

*R/M* recurrent/metastatic, *R* recurrent, *M* metastatic, *NPC* nasopharyngeal carcinoma, *GC* gastric cancer, *R/R* relapsed/refractory, *NHL* non-Hodgkin lymphoma, *HL* Hodgkin lymphoma

NPC is characterized by high expression of PD-L1 (up to 90% of tumor cells) and a large amount of non-malignant lymphocyte infiltration (about 50% of the sample interstitial TIL > 70% or intratumoral TIL > 10%), making NPC patients suitable for PD-1/PD-L1 immune checkpoint blockade therapy.^394–396^ In a phase Ib clinical study, the objective response rate (ORR) for pembrolizumab in patients was 18%, the median PFS was 2 months, and the median OS was 13 months.^397^ For Hodgkin’s lymphoma, immune checkpoint blockade of PD-1 has also shown promising results (NCT01592370).^392^ Gastric cancer is currently classified into four subcategories, including EBV-positive, microsatellite instability (MSI), genomic stability, and chromosomal instability.^29^ In EBVaGC, the virus is cleverly positioned in the infected cells and microenvironment to counteract host immune cells through exosomes whereas cancer cells express PD-L1 and recruit PD-L1-positive immune cells to evade the host immune system.^364^ In a phase II clinical trial of pembrolizumab in metastatic gastric cancer, high MSI and the overall effectiveness rate of EBVaGC patients was 85.7 and 100%, respectively. The presence of EBV was also shown to be a biomarker for pembrolizumab treatment.^398^

Nivolumab was the first PD-1 monoclonal antibody approved for clinical use. The total ORR of 44 patients was 20.5%. The 1-year OS rate was 59%, the 1-year PFS rate was 19.3%, and the patients with high PD-L1 had better response to nivolumab, indicating that nivolumab has a good application prospect in NPC.^399^ PD-1 blockade also in relapsed/refractory B-cell non-Hodgkin’s lymphoma in a phase I trial of nivolumab showed promising results, including follicular lymphomas (which often show abundant PD-1 expression on intratumoral CD4^+^ T cells) and diffuse large B-cell lymphoma (variably expressing PD-1 and PD-L1).^392,400^ In a phase I trial of 27 patients with unresectable or metastatic NPC, these patients failed the previous standard treatment and produced an objective 26% response to pembrolizumab treatment, with a 1-year overall survival rate of 63%. In addition, the efficacy and safety of anti-PD-1 immune checkpoint inhibitors have been demonstrated in numerous clinical trials in recurrent or metastatic EBV-positive tumors. The unusually high response rate in phase I camrelizumab chemotherapy studies suggests that camrelizumab may have shed light on this potentially promising direction,^401^ and the combination of PD-1 inhibitors with other chemotherapeutic agents has also been shown to greatly improve overall survival.^402^ Fang et al^403^ reported that 23 patients with metastatic or recurrent NPC treated with a combination of camrelizumab and gemcitabine/cisplatin, the ORR was 91%, and the 1-year PFS was 61%. Camrelizumab combined with chemotherapy shows good tolerance and antitumor activity, and it is a potential treatment choice for patients with recurrent or metastatic NPC. Therefore, Sun Yat-sen University launched a Phase III clinical trial of camrelizumab combined with gemcitabine and cisplatin (NCT03707509). These results will demonstrate that a PD-1 monoclonal antibody is the first choice to prevent the failure of chemotherapy. Whether the PD-1 monoclonal antibody is better to use alone or in combination as a first-line drug treatment needs to be further explored.

### EBV-related autoimmune diseases (SADs) and immunotherapy

SADs are a group of connective tissue diseases. EBV infection has an etiopathogenetic role in SADs, such as SLE, RA, and SS.^61,404,405^ RA patients have an increased risk of EBV-associated lymphoproliferative disease (LPD), and SS also increases the risk of developing EBV-associated malignancies.^404^ Patients with SADs exhibited EBV-specific T-cell defects, increased viral load, as well as high levels of EBV antibodies.^406–408^ The relationship between EBV and SADs is complex and involves different mechanisms. The proteins encoded by EBV are involved in immune evasion and suppression of apoptosis in transformed infected lymphocytes, leading to loss of immune tolerance and autoimmunity.^409–411^ In addition, molecular mimicry between EBV proteins and autoantigens provides another possible model for disease induction and immune escape.^405^ RA susceptibility is carried by the HLA-DRB1* allele, whose third highly variable region contains the QK/RRAA or RRRAA base sequence, which is known as a shared epitope.^412^ The QKRAA amino acid sequence of HLA-DRB1*04:01 can also be found in the EBV glycoprotein gp110, the molecular mimicry between these EBV epitopes and autoantigens could lead to tolerance and autoimmune system collapse in RA patients.^413^ Sequence similarity between EBNA1 and ANO2 that overlaps with the minimal epitope defined by ANO2 and is known to correlate with MS risk.^405^ This evidence suggested that viral infection may initiate an autoimmune response through molecular mimicry,^414^ thus providing some degree of mechanistic linkage between EBV and SADs.

A marked reduction in interferon gamma-producing CD8^+^ cytotoxic T cells is commonly observed in patients with SADs, which may be the result of defective or reduced EBV-specific cytotoxic T cells, resulting in poor control of EBV infection.^406,415,416^ Thus, cellular immunotherapy is currently one of the most promising treatment options for EBV-related autoimmune diseases.^61^ A phase I clinical trial of specific T cells targeting EBNA1, LMP1, and LMP2A was conducted in five patients with secondary progressive MS and five patients with primary progressive MS.^417^ Further clinical trials will help determine the efficacy of EBV-specific T cells in the treatment of SADs.

### EBV and immune escape

Avoiding immune destruction is an important cancer hallmarker. EBV has developed powerful strategies to evade host immune surveillance, with varying mechanisms of immune escape in EBV-driven malignancies. EBV-associated tumors are often rich in lymphoid-like mesenchyme, and a recent study found EBV-positive was detected in 74.5% of hepatocellular carcinomas with immune cell stroma (HCC-IS).^418–420^ This subtype of hepatocellular carcinoma has high-density EB-associated TIL infiltration and poorer RFS and OS characteristics, which may be related to the upregulation of CD28 co-stimulatory signals, T-cell receptors and other signaling pathways in HCC-IS, leading to CD8^+^ T-cell depletion and thus induced immune escape^420^ in gastric cancer are silenced up to 75% EBVaGC exhibits a high frequency of DNA methylation, thus serving as a strategy for the virus to evade host immune regulation.^421–423^ Immune-related genes (IRGS) in EBVaGC are silenced up to 75% by methylation, such as metallothionein-1 (MT1) and homologous box A (HOXA) clusters, thereby promoting EBV cleavage replication and subviral particle release, as well as KSHV cleavage reactivation.^421^ Collectively,these results demonstrated that epigenetic silencing of IRGs is a viral strategy to escape immune surveillance and promote viral propagation, which is overall beneficial to viral oncogenesis of human gamma-herpesviruses, considering that these IRGs possess antiviral activities against these oncoviruses.^421^

Reports supporting the important role of the tumor microenvironment (TME) in the development of nasopharyngeal carcinoma, which is composed of cellular components and non-cellular ECM (extracellular matrix). A key feature of NPC-TME is the presence of a large number of tumor-infiltrating lymphocytes (TILs) in the NPC tumor stroma, and elevated levels of IL-6, IL-8, IL-10, and interferon-γ and decreased levels of IL-2 were commonly detected in biopsied tissues, which may be related to the recruitment and activity of these infiltrating leukocytes.^424–427^ EBV-encoded Zta can bind to the IL-8 promoter and induce expression of IL-8 by nasopharyngeal carcinoma cells.^428^ IL-8 is known to be highly chemotactic for neutrophils, which are thought to be the first immune cells to be activated and recruited to inflammatory tissues.^429,430^ Zta has also been shown to manipulate neighboring monocytes to release IL-10 into the TME.^431^ Zta upregulates two immunomodulators in nasopharyngeal cancer cells: granulocyte macrophage colony-stimulating factor (GM-CSF) and prostaglandin E2 (PGE2), both of which are released into the TME and have synergistic effects in promoting IL-10 production by monocytes.^431^ IL-10 has long been recognized as a potent immunosuppressive cytokine that effectively inhibits the cytotoxic function of activated CD8^+^ cells and promotes the survival of EBV-infected NPC cells.^432^ Also EBV latent membrane protein 1 (LMP1) induces tyrosine sulfation of CXCR4 via tyrosine protein sulfotransferase-1 (TPST-1), the enzyme responsible for catalyzing tyrosine sulfation in vivo, which may be related to the high metastatic properties of NPC.^433,434^

EBV cleavage replication is involved in the early stages of B-cell lymphangiogenesis by promoting the release of soluble factors from the microenvironment, directly promoting B-cell growth, suppressing the antitumor immune response, and stimulating neovascularization. LMP-1 is a major driver of increased expression and local release of cytokines and chemokines that may be functionally relevant to B-cell growth and survival, such as IL-6 and IL-10.^435^ LMP-2A may also induce B cells to produce more IL-10 through activation of PI3-kinase,^436^ and EBV may also interfere with the host cytokine environment by producing viral IL-10 homologs that activate autocrine signaling pathways involving JAK/STAT.^437^ Alternatively, EBNA1 may favor the formation of an immunosuppressive microenvironment by upregulating CCL20 to attract regulatory T (Treg) cells to the infected tissues.^438^

Evidence accumulated so far clearly indicates that EBV choreographs complex mechanisms that favor immune escape of tumor cells while creating an environment conducive to tumor cell growth and survival. The elucidation of the complex mechanisms between EBV infection and different tumors is one of the future research directions, which will provide powerful therapeutic targets for EBV-driven malignancies.

## Future perspectives

1. EBV research highlights the insights showing that this virus continues to play an important etiological and pathogenetic role in EBV-associated cancers and other diseases. As an epigenetic driver, EBV mediates epigenetic effects, which are closely associated with a methylation-specific phenotype (EBV-CIMP). Also, EBV infection disrupts the balance in DNA methylation or demethylation. Development of panels of methylation genes and noninvasive DNA-based liquid biopsies, which exhibit disease-specific methylation patterns, will provide specific clinical significance for diagnosing these diseases. Discovery of promising demethylation agents for intervention in EBV-CIMP will be a new future direction.
2. Accumulating evidence confirms that EBV can target mitochondria directly or indirectly. The onco-signaling from EBV-encoded oncoproteins alter multiple mitochondrial functions, including metabolic reprogramming, cell death resistance, and mitochondrial dynamics. These finding provide new insights in understanding the role of mitochondria in EBV-associated carcinogenesis. Thus, targeting mitochondrial-related signaling will be a novel therapeutic strategy against EBV-associated diseases.
3. Lytic EBV replication contributes to viral-related tumorigenesis and systemic autoimmune diseases. The association of varying degrees of lytic EBV replication with EBV-related malignancies corresponds directly with the therapeutic resistance and prognosis of patients. Understanding new mechanisms of lytic replication and targeting lytic anti-genes will assist in early identification of high-risk individuals and is very meaningful in clinical settings, including staging and therapeutic responses.
4. EBV co-evolves with the host, which establishes a life-long interacting relationship between the pathogen and host. Interestingly, EBV relies on host machinery and hijacks the ubiquitin system, even encoding-DUB to meet its survival needs. Also, EBV-encoded ncRNA is a highly coordinated mechanism through which EBV might mimic a competition with human miRNAs to affect cellular functions by targeting host genes. Moreover, molecular mimicry between EBV and host proteins builds up a new link between EBV infection and SADs. The ultimate challenge is to translate these scientific findings into novel and more effective diagnosis and clinical therapeutic strategies in the future.
5. EBV has been shown to trigger a strong immune response, which provides a good basis for immunotherapy in EBV-related diseases. Further clinical trials will help determine the immunotherapy in the treatment of these diseases and provide a therapeutic option for patients. In addition, new vaccination strategies against EBV need to be further explored and will be a most important issue for EBV-associated diseases.
